# SiO_2_-*g*-Poly(sulfobetaine methacrylate) Inorganic–Organic Hybrids as Fracturing Fluids Under Harsh Reservoir Conditions

**DOI:** 10.3390/polym18202497

**Published:** 2026-10-12

**Authors:** Cheng Huang, Hongyi Li, Xuefeng Gong

**Affiliations:** 1 School of Energy, China University of Geosciences (Beijing), Beijing 100190, China; 13963370027@163.com (H.L.); wxygxf2005@163.com (X.G.); 2 Shengli Oilfield Oil Development Center Co., Ltd., Dongying 257100, China; 3 Academy of Science and Technology, China University of Petroleum (East China), Dongying 257061, China

**Keywords:** fracture fluid, grafting polymer, inorganic–organic hybrids, rheology, oil recovery

## Abstract

Zwitterionic polymer-grafted inorganic nanoparticles have emerged as promising functional additives for hydraulic fracturing fluids under harsh reservoir conditions, yet achieving precise control over graft chain length and understanding its structure–performance relationship remain challenging. Herein, well-defined SiO_2_-*g*-poly(sulfobetaine methacrylate) (SiO_2_-*g*-PSBMA) inorganic–organic hybrid nanoparticles with tunable chain lengths were synthesized via surface-initiated atom-transfer radical polymerization (SI-ATRP) at 80 °C. The number-average molecular weight (M_n_) of the grafted PSBMA chains was precisely tuned from 3.1 to 14.2 kg mol^−1^ with narrow dispersity (Đ = 1.64–1.78), and the corresponding grafting density was calculated to range from 0.12 to 0.45 chains nm^−2^ by combining TGA, GPC, and BET analyses. Interfacial tension measurements showed that the SiO_2_-*g*-PSBMA_14_k fluid reduced the oil–water interfacial tension from 28 to 5.1 mN m^−1^ at 90 °C and retained effective tension reduction (7.8 mN m^−1^) even at 20 wt% NaCl. Rock-adsorption studies on tight sandstone revealed that longer graft chains achieved a higher saturated adsorption capacity of 2.1 mg g^−1^. Core-flooding experiments using natural Xujiahe tight sandstone cores at 90 °C demonstrated that the SiO_2_-*g*-PSBMA_14_k fracturing fluid at an optimal concentration of 1.0 g L^−1^ achieved ultimate oil recovery (R_oil_) of approximately 41% for low-permeability cores (D = 5.1 × 10^3^ μm^2^) and approximately 52% for higher-permeability cores (D = 112.5 × 10^3^ μm^2^). These results establish the chain-length-dependent structure–performance relationship of SiO_2_-*g*-PSBMA hybrids under simulated reservoir conditions (up to 90 °C and 20 wt% NaCl) and provide guidance for the rational design of zwitterionic nanofluid additives for fracturing applications.

## 1. Introduction

With the increasing demand of global energy, especially oil and gas resources, the exploitation of unconventional hydrocarbons has become a strategic focus in the petroleum industry [1,2,3]. Hydraulic fracturing, as a pivotal stimulation technology, creates complex fracture networks in low-permeability reservoirs to enhance hydrocarbon flow pathways and achieve commercial production [4,5]. However, deep and ultra-deep tight reservoirs, such as the Xujiahe Formation in the Sichuan Basin, present uniquely harsh environments characterized by burial depths exceeding 4500 m, elevated temperatures above 120 °C, abnormal high pore pressures, and high-salinity formation brines [6,7]. Under three high conditions, including high temperature, pressure, and salinity, conventional fracturing fluids often suffer severe performance degradation, including thermal decomposition, viscosity loss, and chain collapse, leading to inadequate proppant transport, formation damage, and low flowback efficiency [8,9,10]. Currently, the industry-standard fracturing fluids are primarily based on natural polymers and their derivatives, such as guar gum, hydroxypropyl guar (HPG), and carboxymethyl hydroxypropyl guar (CMHPG) [11,12,13]. Although guar-based fluids offer cost-effectiveness and high initial viscosity, their glycosidic bonds are prone to hydrolytic cleavage at temperatures above 120 °C, while high salinity induces chain coiling and dramatic viscosity reduction [14,15]. HPG exhibits improved thermal stability up to 150 °C and produces less residue (3%) compared to guar gum (13%), yet its tolerance to divalent cations remains limited [16]. CMHPG can withstand temperatures up to 177 °C while tolerating high salinities, but its higher cost and complex crosslinking chemistry restrict widespread application [17]. Synthetic polymers, including hydrophobically associating polyacrylamide (HAPAM) and partially hydrolyzed polyacrylamide (HPAM), have emerged as alternatives. However, HPAM is highly sensitive to temperature and shear stress, exhibiting a significant reduction in rheological stability at high temperatures [18,19]. Viscoelastic surfactant (VES)-based fluids, while offering clean breaking properties, suffer from low thermal stability to form effective filter cakes, resulting in excessive fluid loss [20]. The promoted salt tolerance and thermal stability of fracturing fluids still face a great challenge of deep shale gas and tight sandstone development [21,22].

To address these limitations, the integration of nanotechnology with polymer chemistry has a new inspiration to design the next-generation fracturing fluids [23,24]. Nanoparticle-reinforced polymer systems, particularly silica (SiO_2_)-based nanohybrids, have demonstrated remarkable potential in improving rheological properties, reducing interfacial tension, and altering wettability under harsh reservoir conditions [25,26]. The introduction of SiO_2_ nanoparticles into polymer matrices enhances viscosity through the formation of dense network structures via electrostatic and hydrogen-bonding interactions, while simultaneously reducing polymer adsorption on rock surfaces by up to 50% [27,28]. However, most existing studies rely on physical blending of nanoparticles and polymers, which suffers from inherent drawbacks: nanoparticles tend to aggregate in high-salinity brines due to suppressed electrostatic repulsion, and physically adsorbed polymer chains are susceptible to detachment under high shear and elevated temperatures, leading to phase separation and compromised long-term stability [29,30]. In contrast, covalently grafted polymer chains onto inorganic–organic interfaces offer a robust strategy to suppress chain dissociation and precisely control the topological architecture of the resulting hybrid materials [31,32]. Among various polymer architectures, zwitterionic polymers featuring both cations and anions on the same monomer unit have attracted considerable attention for their unique “anti-polyelectrolyte effect” [33,34,35]. Unlike conventional polyelectrolytes that exhibit chain collapse with increasing ionic strength, zwitterionic polymers such as poly(sulfobetaine methacrylate) (PSBMA) demonstrate chain extension and enhanced hydrodynamic volume in saline environments due to the disruption of intrachain dipole–dipole pairing by salt counterions [36,37]. This intrinsic salt-thickening behavior endows zwitterionic fluids with superior viscosity retention in high-salinity environments, offering a novel solution for application in high-temperature/high-salinity (HTHS) reservoirs [38,39]. Recent studies have shown that zwitterionic polymers can maintain over 70% of saturated viscosity in flowback fluids with 10,000 mg L^−1^ of Ca^2+^, significantly outperforming conventional sulfobetaine-based polymers [40]. Furthermore, the dense hydration layer formed by zwitterionic moieties can bind up to plenty of water molecules, markedly improving competitive hydration resistance against metal ions [41].

The precise synthesis of well-defined polymer–nanoparticle hybrids with tunable chain lengths and graft densities is essential for establishing quantitative structure–property relationships and optimizing fracturing fluid performance [42]. Surface-initiated atom transfer radical polymerization (SI-ATRP) has emerged as a powerful controlled/living radical polymerization technique for fabricating such architectures [43,44]. Originating from conventional ATRP, SI-ATRP extends the reversible redox mechanism to interfaces, enabling the growth of grafted chains directly from immobilized initiator sites [45,46]. This method offers exceptional control for adjustable molecular weight and grafting densities, which are difficult to achieve through conventional free-radical polymerization or physical blending methods [47,48]. By simply adjusting the nanoparticle feeding ratio or polymerization time, the degree of polymerization and brush thickness can be systematically tuned from several nanometers to over 100 nm, providing critical structural flexibility for tailoring interfacial activity, rock-adsorption behavior, and bulk rheological properties [49,50]. Despite the promising attributes of zwitterionic polymers and SiO_2_-based nanohybrids, few studies have systematically investigated the combined advantages of covalently grafted zwitterionic brushes on silica nanoparticles for fracturing fluid applications under HTHS conditions. Previous reports on polymer-coated silica nanoparticles for enhanced oil recovery (EOR) have demonstrated incremental oil recoveries ranging from 2.6% to 17% of original oil in place (OOIP) in tertiary flooding mode, with secondary recoveries reaching up to 15 percentage points above waterflood baselines [49,50]. Core-flooding experiments using SiO_2_–polymer hybrids at 90 °C achieved cumulative oil recoveries of approximately 60.8% OOIP, outperforming conventional HPAM (54.7% OOIP) under identical conditions [51,52,53]. However, these systems were primarily designed for EOR flooding rather than fracturing applications, and the precise modulation of grafted chain lengths to optimize interfacial tension reduction, rock-adsorption kinetics, and proppant transport under extreme salinities (up to 20 wt % NaCl) remains largely unexplored.

Herein, we report the design, synthesis, and comprehensive evaluation of well-defined SiO_2_-*g*-poly(sulfobetaine methacrylate) (SiO_2_-*g*-PSBMA) inorganic–organic hybrid nanoparticles with tunable graft chain lengths via SI-ATRP for application as fracturing fluids under harsh reservoir conditions. The covalent grafting architecture was specifically designed to suppress chain detachment under high temperature and high salinity, while the zwitterionic PSBMA segments provide intrinsic salt tolerance through the anti-polyelectrolyte effect. By systematically varying the silica nano-initiator feeding ratio, the number-average molecular weight (M_n_) of grafted PSBMA chains was precisely tuned from 3.1 to 14.2 kg·mol^−1^ with narrow dispersity (PDI = 1.64–1.78). Detailed characterizations confirmed the successful immobilization of zwitterionic segments and revealed chain-length-dependent self-assembly behaviors governed by interchain dipole–dipole interactions. Rheological evaluations demonstrated favorable shear-thinning characteristics, moderate thermal stability up to 90 °C, and reasonable salt tolerance in both NaCl and CaCl_2_ environments. Interfacial tension measurements and rock-adsorption kinetic studies on the tight sandstone matrix established that longer PSBMA graft chains contribute to higher saturated adsorption capacity and favorable oil–water interfacial activity, with the nanofluid retaining effective tension reduction even at 20 wt% of NaCl. Core-flooding experiments using natural Xujiahe tight sandstone cores at 90 °C demonstrated that the SiO_2_-*g*-PSBMA_14k_ fracturing fluid achieves desirable enhanced oil recovery under moderate nanoparticle loading, with performance governed by injection velocity, concentration, and pore-throat dimensions. This work provides a controllable synthetic strategy for zwitterionic hybrid additives and clarifies chain-length-dependent mechanisms under harsh reservoir environments, offering a promising alternative to conventional fracturing fluids for deep tight sandstone gas reservoirs.

## 2. Design of Experiments

### 2.1. Materials

2-bromoisobutyric acid, 3-aminopropyltriethoxysilane (APTES), copper(I) bromide (CuBr), copper(II) bromide (CuBr_2_), 2,2′ bipyridine (bipy), sodium chloride (NaCl), tetrahydrofuran (THF), methanol, ethanol, toluene and n hexane were purchased from Macklin Biochemical Co., Ltd. (Shanghai, China), which were purified by column chromatography before use. Zwitterionic monomer sulfobetaine methacrylate (SBMA, 98%) was obtained from Sigma Aldrich (Shanghai, China). Spherical nano silica (SiO_2_ NP average diameter = 20 nm) was supplied by XFNANO Materials Tech Co., Ltd. (Nanjing, China) Simulated formation brine was prepared according to the ion composition of Sichuan Xujiahe tight sandstone reservoir water. Natural tight sandstone core plugs were machined from Xujiahe formation outcrop, with dimensions of 25 mm diameter × 50 mm length. All of the aqueous solutions were prepared by using deionized water.

### 2.2. Synthesis of SiO_2_-Br Initiator

Prior to grafting, silica nanoparticles were dried under vacuum at 110 °C for 12 h to remove moisture. A measurement of 2.0 g of dried SiO_2_ NP was dispersed in 150 mL anhydrous toluene under vigorous nitrogen stirring, followed by drop-wise addition of 4.0 mL of APTES. The reaction proceeded at 110 °C under N_2_ atmosphere for 24 h. APTES-modified amino-functionalized silica was collected by centrifugation, washed by ethanol for 5 times to remove unreacted silane reagent, and vacuum-dried at 60 °C. Afterwards, amino-modified silica was redispersed in 120 mL anhydrous THF, 3.2 mL of 2-bromoisobutyric acid was added for amidation at room temperature within 18 h. The bromine-terminated SI-ATRP initiator SiO_2_-Br was isolated via centrifugation, thoroughly rinsed with THF and methanol, and vacuum-dried at 45 °C overnight.

### 2.3. SI-ATRP Synthesis of SiO_2_-g-PSBMA Hybrid Nanoparticles

SiO_2_-*g*-PSBMA with varied grafted molecular weights were synthesized via surface-initiated ATRP. Typically, 0.50 g of SiO_2_-Br solid was dispersed in 80 mL mixed solvent (methanol/deionized water = 1:1, *v*/*v*) inside a Schlenk flask (Ruixiang Co., Ltd., Beijing, China). SBMA monomer, CuBr, CuBr_2_ and 2,2′-bipyridine were sequentially added according to predetermined molar ratios. After three cycles of freeze–pump–thaw to eliminate dissolved O_2_, the SI-ATRP was carried out at 80 °C under N_2_ protection. Different reaction durations were adopted to adjust grafting chain length. The reaction mixture was exposed to air to terminate radical propagation. Crude products were purified by repeated centrifugation–redispersion cycles using methanol–water mixed solvent to eliminate residual monomer, free PSBMA homopolymer and copper-based catalyst complexes. The final SiO_2_-*g*-PSBMA hybrid nanoparticles were dried in vacuum at 50 °C for 24 h.

### 2.4. Structural Characterizations

Fourier-transform infrared spectroscopy (FTIR): FTIR spectra were recorded on a Thermo Scientific Nicolet iS50 spectrometer, according to a previous report [51].

Proton nuclear magnetic resonance spectroscopy (^1^H NMR): Free PSBMA chains were cleaved from silica surface using excess tetra-n-butylammonium fluoride (TBAF) in H_2_O. ^1^H NMR measurements were performed according to a previous report [36,37].

Gel permeation chromatography (GPC): Free PSBMA chains were cleaved from the silica surface using excess tetra-n-butylammonium fluoride (TBAF) in deionized water at 50 °C for 12 h. Mn and PDI of cleaved PSBMA chains were determined by an Agilent 1260 Infinity II GPC system using 1,1,1,3,3,3-hexafluoroisopropanol (HFIP) as the mobile phase at a flow rate of 1.0 mL min^−1^ and 35 °C, calibrated with narrow-dispersity poly(methyl methacrylate) (PMMA) standards.

TBAF cleavage verification method: After TBAF treatment, the residual silica solid was collected by centrifugation, washed, and dried. FTIR spectra were recorded to monitor the disappearance of PSBMA characteristic absorption bands (C=O stretching at 1670 cm^−1^ and S=O stretching at 1035 cm^−1^) relative to the invariant silica Si-O-Si peak at 1110 cm^−1^. Complete disappearance of PSBMA peaks in the residual silica confirms quantitative detachment of all grafted chains.

Dynamic light scattering (DLS): Hydrodynamic particle size and size distribution of SiO_2_-*g*-PSBMA nanoparticles were measured by a Malvern Zetasizer Nano ZS instrument (Malvern Co., Ltd., Zhuhai, China). Fluid samples were diluted to 1 g L^−1^ with deionized water or high-salinity brine prior to measurement. Testing temperature was set at 25 °C, and each sample was subjected to three repeated measurements to obtain averaged hydrodynamic diameter and polydispersity index (PDI).

Thermogravimetric analysis (TGA): Thermal decomposition profiles were recorded on a Netzsch STA 449 F3 thermogravimetric analyzer (NETZSCH Co., Ltd., Berlin, German), according to references [37].

X-ray diffraction (XRD): X-ray diffraction patterns were collected on a Bruker D8 Advance X-ray diffractometer equipped with CuKα radiation (λ = 0.15418 nm), according to references [36,37].

Low-field nuclear magnetic resonance (LF-NMR): Low-field NMR (T_2_) relaxation measurements were performed on a MesoMR23-060V-I LF-NMR analyzer (CQITEC Co., Ltd., Hefei, China). Crushed tight sandstone rock powders were saturated with SiO_2_-*g*-PSBMA fluid (1 g L^−1^) at 90 °C. The Carr–Purcell–Meiboom–Gill (CPMG) pulse sequence was adopted to acquire transverse relaxation time (T_2_) spectra. Echo time was set to 0.1 ms, number of echoes was 12,000, and the obtained T_2_ distribution reflected the pore-water state variation after nanoparticle adsorption on rock surfaces.

Rotational rheology (viscosity measurement): Steady-state rheological behaviors of SiO_2_-*g*-PSBMA fluids were determined using a TA Instruments DHR-2 rotational rheometer. Nanoparticle aqueous solutions were prepared at a series of concentrations. Steady shear tests were carried out over a shearing range of 0.1~1000 s^−1^. Test temperature was controlled at 90 °C to simulate reservoir conditions. Viscosity–shear rate curves were recorded to analyze shear-thinning characteristics, fitted by the power-law model for flow-behavior index (*n*) and consistency coefficient (*K*).

Interfacial tension measurement: Dynamic oil–water interfacial tension (*γ*_i_) was measured using a DSA100 pendant-drop tensiometer (Krüss Co., Ltd., Berlin, German). SiO_2_-*g*-PSBMA_14k_ aqueous solution was fixed at 1 g L^−1^. Test temperatures were controlled ranging from 40 to 90 °C, respectively. For salinity-dependent tests (Figure S9), NaCl mass fractions were adjusted from 10 to 20 wt%, and all salinity series were measured at 90 °C. Interfacial tension values were continuously recorded within 45 min for each parallel sample.

### 2.5. Static Rock-Adsorption Experiments

Adsorption performances of SiO_2_-*g*-PSBMA variants on crushed dry Xujiahe tight sandstone core powder were evaluated at 90 °C. For concentration-dependent adsorption, nanoparticle concentration (*c*_NP_) ranged from 0 to 1100 mg L^−1^. For adsorption-kinetic experiments, *c*_NP_ was fixed at 1 g L^−1^, and adsorption time varied from 0 min to 60 min. After reaching preset adsorption time, solid–liquid separation was implemented by high-speed centrifugation. The residual nanoparticle concentration was analyzed via UV-Vis spectrophotometer (Shimadzu UV-2600, Jinpeng Co., Ltd., Shanghai, China), and adsorbed mass per unit rock mass was calculated by mass-difference method.

### 2.6. Core-Flooding Experiments

Core-flooding displacement tests were performed on a homemade high-temperature high-pressure core flooding system. Natural Xujiahe tight sandstone core plugs were cleaned with toluene–methanol mixed solvent, dried under vacuum, and then saturated with crude oil to establish initial oil-saturation state. The whole experimental temperature was maintained at 90 °C. The standard displacement sequence followed pre-water flooding, fluid flooding and post-water flooding. For concentration-gradient tests, nanoparticle concentrations *c*_NP_ were set as 0.5, 1.0 and 3.0 g L^−1^ with the injection flow velocity of 0.20 mL min^−1^. For pore-throat effect evaluation, cores with different average pore-throat dimension *D* were adopted at *c*_NP_ of 1 g L^−1^. For injection-velocity series, flow velocities were adjusted to 0.05, 0.10 and 0.20 mL min^−1^ with constant *c*_NP_ of 1 g L^−1^. Real-time water content, injection pressure and oil recovery ratio were continuously recorded as a function of injected pore volume. Each experimental group was conducted with at least two parallel core samples.

Note: All quantitative measurements (DLS, rheology, interfacial tension, adsorption, and core flooding) were performed with at least three independent samples (*n* = 3). Data are presented as mean ± standard deviation (S.D.). Error bars in all figures represent one standard deviation. Core-flooding experiments were conducted with at least two parallel core samples per condition.

## 3. Results and Discussions

### 3.1. Synthesis and Structure of SiO_2_-g-PSBMA Grafting Polymers

**Scheme 1:**
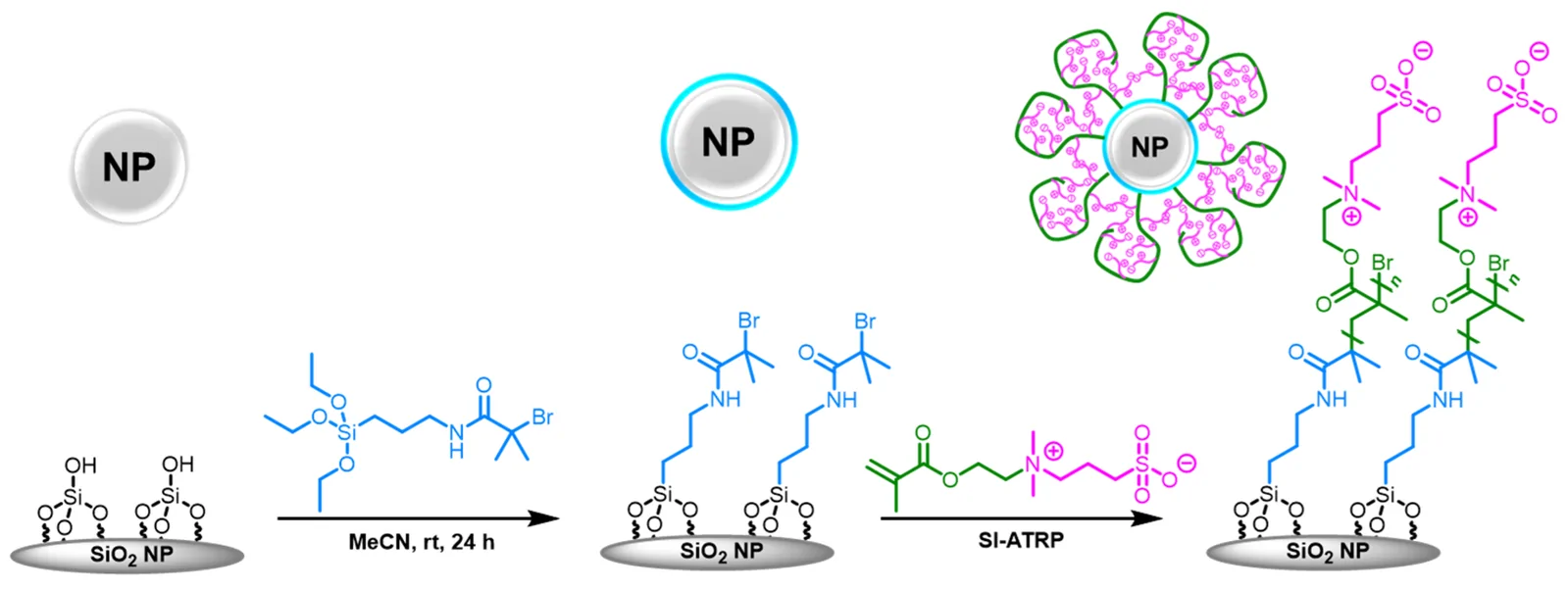
Synthesis, chemical and topological structure of SiO_2_-*g*-PSBMA grafting polymer hybrid materials, synthesized by SI-ATRP.

Surface-initiated atom transfer radical polymerization (SI-ATRP) was employed to fabricate zwitterionic SiO_2_-*g*-PSBMA organic–inorganic hybrid materials, and the synthetic procedure is illustrated in Scheme 1. Briefly, bare silica nanoparticles (SiO_2_ NPs) with abundant surface silanol groups were first modified with 3-aminopropyltriethoxysilane (APTES) in anhydrous toluene at 110 °C under N_2_ atmosphere for 24 h, yielding amino-functionalized SiO_2_. Subsequently, 2-bromoisobutyric acid was coupled to the amino groups via amidation in anhydrous THF at room temperature for 18 h to obtain bromine-terminated SI-ATRP initiators (SiO_2_-Br). Sulfobetaine methacrylate (SBMA) monomers then underwent SI-ATRP from the immobilized bromine initiator sites at 80 °C under N_2_ atmosphere to covalently graft PSBMA chains onto silica nanoparticle surfaces. The as-prepared SiO_2_-*g*-PSBMA hybrid products were purified by repeated centrifugation–redispersion cycles to remove residual-free PSBMA homopolymers, unreacted SBMA monomers and catalyst residues before further characterizations. Scheme 1 schematically demonstrates the topological architecture of final hybrids, where dense zwitterionic PSBMA chains are tethered onto spherical SiO_2_ nanoparticle surfaces.

**Figure 1:**
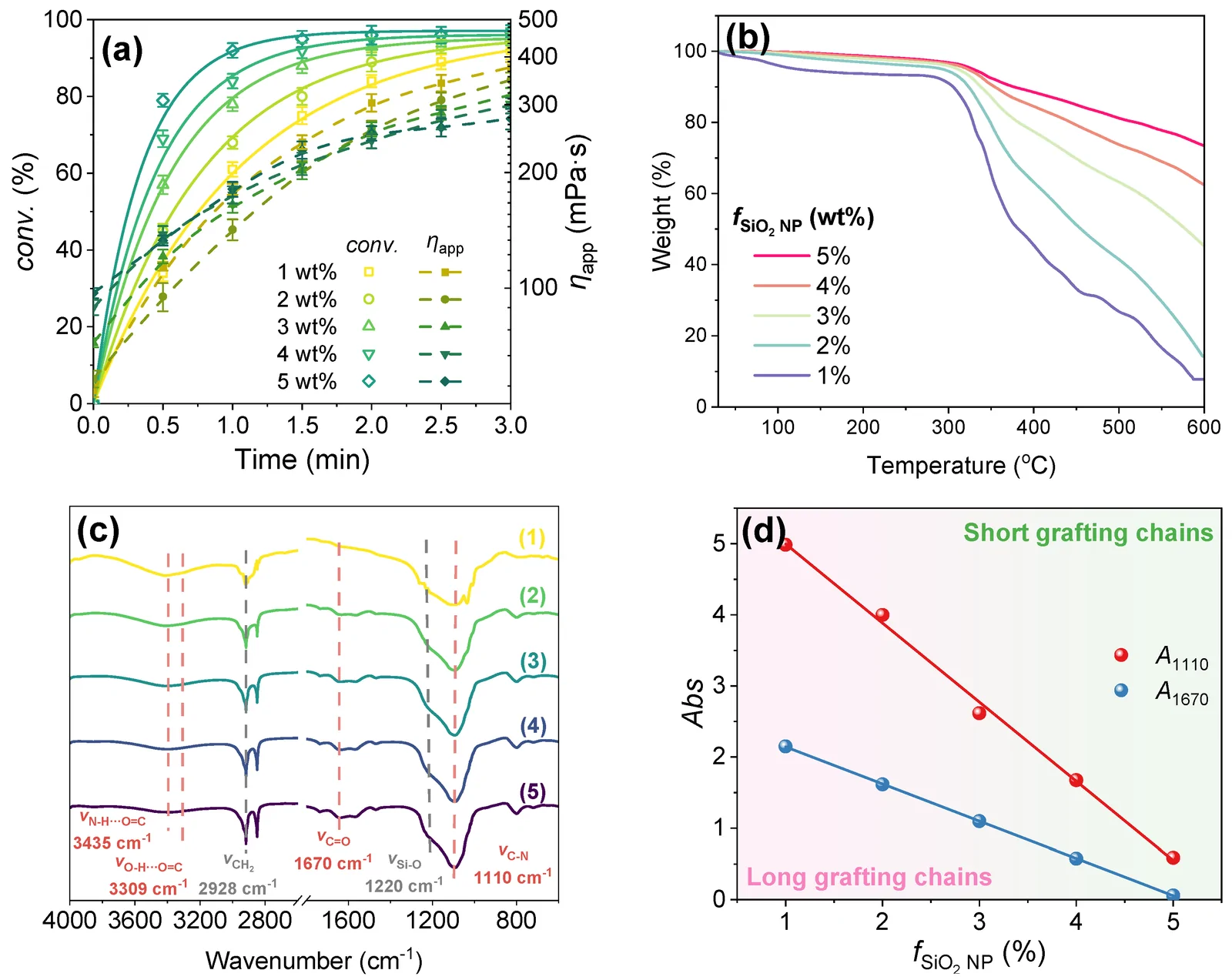
(**a**) Plots of monomer conversion (*conv.*) or apparent viscosity (*η*_app_) vs. polymerization time in different polymerization solution with various original SiO_2_ feeding (*f*_SiO2 NP_). Conditions: 80 °C. (**b**) TGA curves of resulting SiO_2_-PSBMA polymers with various *f*_SiO2 NP_ under N_2_ atmosphere. (**c**) FTIR spectra of resulting SiO_2_-PSBMA polymers with *f*_SiO2 NP_ of (1) 1 wt%, (2) 2 wt%, (3) 3 wt%, (4) 4 wt% and (5) 5 wt%. (**d**) Relationships between absorbances at 1110 or 1670 cm^−1^ (*A*_1110_ or *A*_1670_) and *f*_SiO2 NP_.

The kinetic measurement was carried out to reveal the impact of introduced silica nanoparticles on SBMA polymerization kinetics and the increasing apparent viscosity of polymerization solution systems. Figure 1a presents the time-dependent monomer conversion and apparent viscosity profiles recorded during SI-ATRP polymerization under 80 °C with varied SiO_2_ feedings (*f*_SiO2 NP_) ranging from 1 wt% to 5 wt%. For all tested groups, both monomer conversion and apparent viscosity continuously increased as polymerization proceeded. Higher initial SiO_2_ nanoparticle loading brought about elevated monomer conversion rates as well as higher apparent viscosity values within the monitored reaction window, indicating that the presence of silica nano-cores facilitated the SI-ATRP process and thickened the polymerizing solution.

Thermogravimetric analysis (TGA) was employed to quantify the organic content of the SiO_2_-*g-*PSBMA hybrids (Figure 1b). All samples displayed a small weight loss below 150 °C attributable to desorption of physically adsorbed water, followed by a pronounced two-step decomposition of the grafted PSBMA chains between 300 and 600 °C. The first, more rapid step (300–450 °C) corresponds to random chain scission of the PSBMA backbone and cleavage of the sulfobetaine side groups, while the second, slower step (450~600 °C) reflects decomposition of the thermally more stable carbonaceous char. At 600 °C, the residual inorganic SiO_2_ fractions were approximately 7%, 13%, 45%, 62%, and 73% for *f*_SiO2 NP_ = 1, 2, 3, 4, and 5 wt%, respectively. The monotonic increase in residual weight with increasing *f*_SiO2 NP_ confirms that a higher silica-initiator loading yields a lower mass fraction of grafted PSBMA in the final hybrid, consistent with the GPC results showing shorter chain lengths at higher *f*_SiO2 NP_. To further verify the successful initiator modification and polymer grafting process, comparative thermogravimetric measurements of pristine SiO_2_ nanoparticles, initiator-modified SiO_2_ nano-initiators, neat PSBMA_11k_ homopolymers and SiO_2_-PSBMA hybrids were performed under nitrogen atmosphere, as presented in Figure S1. Bare SiO_2_ NPs exhibited negligible mass loss over the whole temperature range, whereas modified SiO_2_ NPs displayed slight weight loss originating from the decomposition of covalently anchored ATRP initiator moieties. Distinct major weight loss domains of pure PSBMA homopolymers and SiO_2_-PSBMA hybrids were both observed within 250~600 °C; the residual char yield of SiO_2_-PSBMA was significantly higher than that of neat PSBMA, unambiguously confirming the covalent attachment of PSBMA chains onto inorganic silica nanoparticle surfaces.

To verify successful grafting of PSBMA segments onto silica nanoparticles, FTIR analysis was utilized for SiO_2_-*g*-PSBMA samples with *f*_SiO2 NP_ of 1~5 wt%, as shown in Figure 1c. Characteristic absorption bands at 1220 cm^−1^ belonged to Si-O-Si asymmetric stretching of the silica framework (*ν*_Si-O_) from the silica inorganic framework, while the peaks centered at 1110 and 1670 cm^−1^ were assigned to N-H and carbonyl stretching vibration (*ν*_N-H_, *ν*_C=O_) of the PSBMA methacrylate backbone. The broad absorption band near 3309 cm^−1^ is attributed to O-H stretching vibrations of surface silanol groups (ν_O-H_) and hydrogen-bonded N-H···O=C interactions, while the peak at 1670 cm^−1^ corresponds to C=O stretching vibrations (ν_C=O_) of the PSBMA methacrylate backbone, and the absorption at 1110 cm^−1^ is assigned to N-H stretching. The broad absorption near 3435 cm^−1^ was attributed to hydrogen bonds between N-H and C=O stretching vibrations (*ν*_C=O···H-N_), and the vibration signal at 2928 cm^−1^ corresponded to aliphatic C-H stretching (*ν*_CH2_) from PSBMA side-chains [36,37]. Complementary FTIR characterizations displayed in Figure S2a further track the chemical evolution from bare silica nanoparticles and initiator-grafted silica nano-initiators to neat PSBMA homopolymers. The pristine SiO_2_ NPs exhibited the characteristic strong *ν*_N-H_ at 1110 cm^−1^, while initiator-modified SiO_2_ NPs introduced weak carbonyl-related signals inherited from the amide-containing ATRP initiator. Distinctive *ν*_C=O_ at 1670 cm^−1^ originating from methacrylate groups was clearly observed for neat PSBMA_11k_, which serves as the diagnostic fingerprint to identify successful PSBMA grafting on nanoparticle surfaces in the main-text samples. The appearance of PSBMA-related characteristic peaks collectively validated the successful immobilization of zwitterionic polymer chains on SiO_2_ surfaces.

To quantitatively assess the relative grafting chain length under different silica feeding ratios, absorbance values at 1110 cm^−1^ (*A*_1110_, assigned to *ν*_N-H_) and 1670 cm^−1^ (*A*_1670_, assigned to *ν*_C=O_) of the organic PSBMA marker were extracted and plotted against *f*_SiO2 NP_ in Figure 1d. Both *A*_1110_ and *A*_1670_ displayed monotonic decreasing trends upon increasing silica nanoparticle feeding content. With the increment of SiO_2_ loading, the relative length of grafted PSBMA chains became shorter, transitioning from a long grafting-chain regime to short grafting-chain regime. Collectively, these characterization results demonstrated that the grafting chain length of PSBMA could be facilely tuned by adjusting the feeding amount of silica nano-initiators during SI-ATRP, which provides feasible structural regulation for subsequent fracturing-fluid performance investigation.

**Figure 2:**
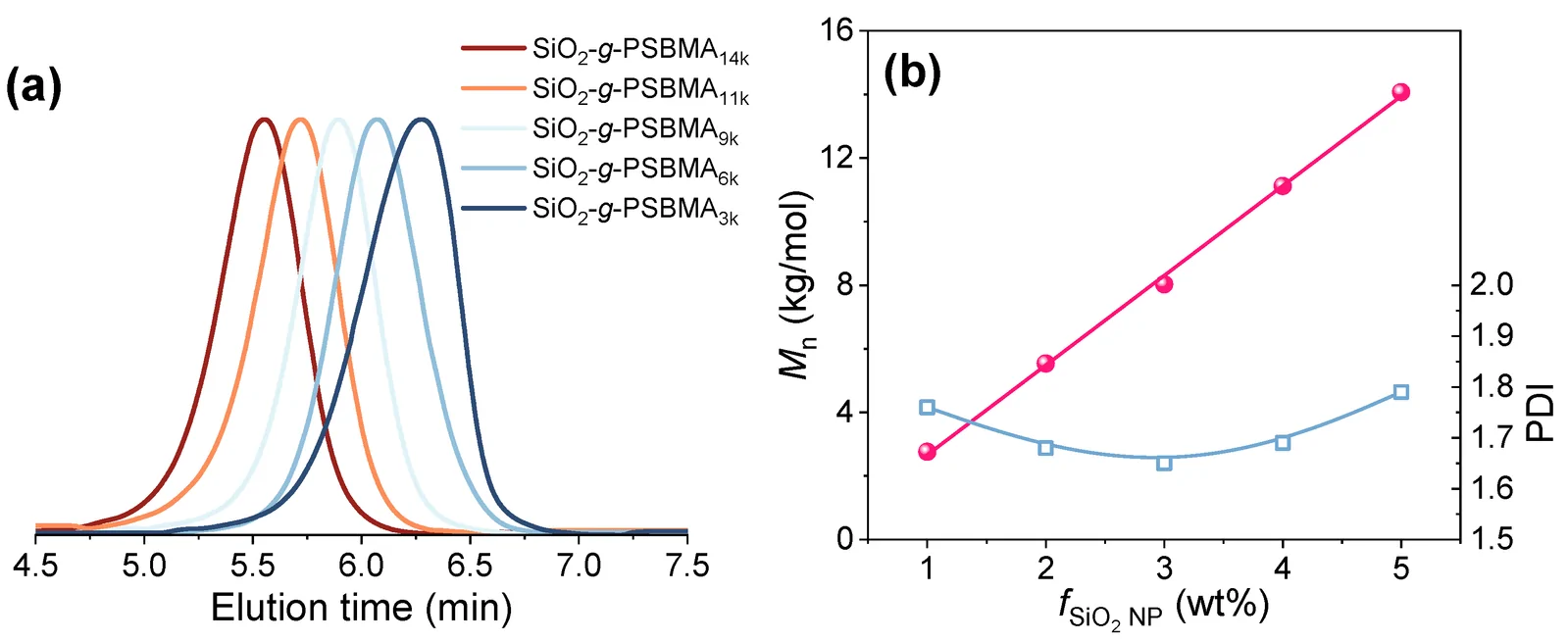

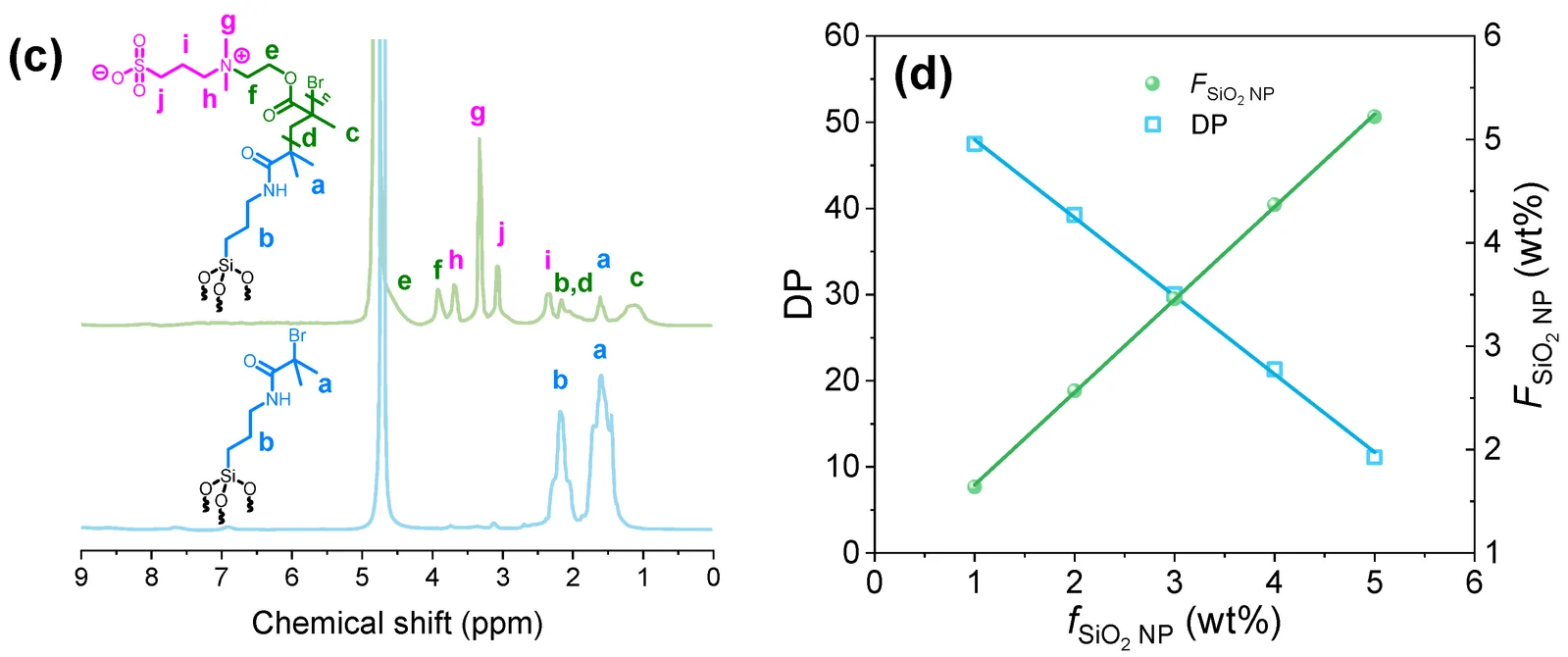
(**a**) GPC traces of residual PSBMA polymers in HFIP cleaved from SiO_2_-*g*-PSBMA grafting polymers by TBAF reagent, calibrated by standard PEO samples. (**b**) Effects of *f*_SiO2 NP_ on *M*_n_ (Red cycles) and PDI (Blue squares). (**c**) Representative ^1^H NMR spectra of modified SiO_2_ and SiO_2_-*g*-PSBMA_3k_ in D_2_O. (**d**) Effects of *f*_SiO2 NP_ on degree of polymerization (DP) and mass content of SiO_2_ in resulting polymers (*F*_SiO2 NP_).

Following the successful verification of chemical grafting, GPC characterization was further utilized to determine the molecular weight and distribution of PSBMA chains covalently tethered on SiO_2_-*g*-PSBMA hybrids. Direct GPC measurement of as-synthesized intact SiO_2_-*g*-PSBMA hybrid nanoparticles is infeasible, since inorganic silica cores would cause irreversible adsorption and column fouling within the GPC system. To circumvent this limitation, tetra-n-butylammonium fluoride (TBAF)-mediated selective cleavage was performed in deionized water at 50 °C for 12 h, verified by FTIR monitoring (Figure S2b). Under these conditions, fluoride anions from TBAF specifically break Si-O-Si covalent linkages at the nanoparticle surface to quantitatively release grafted PSBMA chains into solutions, while the carbon–carbon backbone and zwitterionic side groups of PSBMA remain intact. Silica residues were subsequently removed by centrifugation, and the recovered PSBMA was precipitated, dried and redissolved for subsequent HFIP-phase GPC analysis calibrated against narrow-dispersity PMMA reference standards. Figure 2a presents the GPC elution traces of TBAF-cleaved PSBMA recovered from hybrid samples prepared at varied silica nanoparticle feeding ratios [36]. All chromatograms show well-defined unimodal elution peaks without obvious high-molecular-weight shoulders or low-molecular-weight tailing. As the feeding fraction of SiO_2_ nano-initiators decreases, the elution time shifts toward shorter retention values, which corresponds to a higher molecular weight of the cleaved PSBMA grafts. The corresponding number-average molecular weight (M_n_) and dispersity (PDI) derived from GPC curves are summarized in Figure 2b. As f_SiO2 NP_ increased from 1 to 5 wt%, the M_n_ of cleaved PSBMA chains decreased gradually from 14.2 down to 3.1 kg mol^−1^. Meanwhile, PDI fluctuated within a narrow range of 1.64–1.78 across the entire feeding gradient, without obvious broadening. This relatively low dispersity confirms that the SI-ATRP polymerization maintained good controllability across the whole investigated nano-initiator loading range. This relatively low dispersity confirms that the SI-ATRP polymerization maintained good controllability across the whole investigated nano-initiator loading range.

^1^H NMR measurements using D_2_O as deuterated solvent were performed to validate the chemical structures of initiator-modified silica nano-initiators and final SiO_2_-*g*-PSBMA hybrids, as displayed in Figure 2c. For initiator-modified SiO_2_, proton resonances **a** at 0.8–1.2 ppm (tert-butyl-Br methyl) and **b** at 1.4–1.8 ppm (propyl methylene of silane linker) belonged to the alkyl segments of SI-ATRP initiators. After SI-ATRP grafting reaction, a series of new diagnostic resonance peaks belonging to sulfobetaine zwitterionic side groups of PSBMA segments appeared distinctly: peak **e** at 2.2–2.6 ppm (-C**H**_2_-SO_3_^−^), peak **i** at 2.8–3.1 ppm (-N^+^(C**H**_3_)_2_-), peak **j** 3.3–3.6 ppm (-C**H**_2_-N^+^-), and peak **g** at 4.0–4.3 ppm (-O-C**H**_2_-), together with backbone aliphatic signals (peak **f**), according to references [36,37]. The complete set of PSBMA characteristic signals offered direct spectroscopic evidence for successful formation of surface-tethered zwitterionic polymer chains. Based on quantitative integration of NMR signals together with GPC molecular-weight data, the degree of polymerization (DP) of grafted PSBMA and the actual mass fraction of silica *F*_SiO2 NP_ inside hybrid materials were calculated and plotted against initial silica feeding fractions in Figure 2d. A clear inverse relationship can be observed between DP and nano-initiator feeding content: DP dropped from 48 down to 11 as *f*_SiO2 NP_ increased from 1 to 5 wt%, demonstrating that higher loading of silica-bound initiator sites led to reduced DP for individual grafted PSBMA chains. Meanwhile, the real silica mass fraction within the hybrid increased monotonically from 1.8 to 5.1 wt% with increasing initial SiO_2_ feeding proportion. These molecular-scale characterizations collectively demonstrate that both grafted PSBMA chain lengths and inorganic–organic composition ratios of SiO_2_-*g*-PSBMA hybrids can be precisely tuned by simply adjusting the feeding amount of silica-supported ATRP nano-initiators, which provides critical structural flexibility for tailoring the macroscopic rheological performance required for fracturing-fluid applications.

To ensure complete removal of free and physically adsorbed PSBMA, the crude product was purified by at least five repeated centrifugation–redispersion cycles using methanol–water mixed solvents. The supernatant from each washing step was monitored by FTIR spectroscopy at the PSBMA characteristic absorption wavelength; the absorbance reached baseline after the third cycle, confirming quantitative removal of unbound polymers. The covalent attachment was established by multiple complementary lines of evidence: (i) FTIR spectra of purified hybrids show persistent PSBMA characteristic peaks (1670 cm^−1^ for C=O, 1035 cm^−1^ for S=O) after exhaustive washing, which would not remain for physically adsorbed polymer; (ii) TGA (Figure S1) shows that the hybrid’s decomposition profile differs from a physical mixture of SiO_2_ and PSBMA, with higher residual char yield indicating covalent tethering; (iii) TBAF-mediated cleavage of Si-O-Si bonds quantitatively releases PSBMA chains suitable for GPC analysis, confirming that chains are anchored to the silica surface via covalent siloxane linkages rather than physical adsorption; (iv) ^1^H NMR of cleaved chains shows the complete PSBMA proton signature, confirming intact polymer structure after detachment.

The grafting density (σ) of PSBMA chains on the SiO_2_ surface was quantitatively determined by combining the inorganic mass fraction with GPC-measured M_n_ of TBAF-cleaved chains. The number of chains per nanoparticle was calculated as

*N*_chains_ = (*w_PSBMA_* · *N_A_*/*M_n_*)/(*w*_*SiO*_2__/*m_p_*)

where *w_PSBMA_* = 1 − *w_SiO_*_2_, *N_A_* is Avogadro’s number, and *m_p_* = 9.22 × 10^−18^ *g* is the mass of a single 20 nm SiO_2_ particle (*ρ =* 2.2 g cm^−3^). The grafting density was obtained as *σ = N_chains_/A*_p_, where *A_p_ =* 4*πr*^2^ = 1257 nm^2^.

Two independent measurements of w_SiO2_ were compared: the TGA residual at 600 °C and the F_SiO2_ values from Figure 2d. As summarized in Table 1, the TGA-based grafting densities range from 0.46 to 4.13 chains nm^−2^. For the high-silica samples (f_SiO2_ = 3–5 wt%), σ = 0.46–0.63 chains nm^−2^ falls within the typical polymer-brush regime. The F_SiO2_-based values (14.4–26.5 chains nm^−2^) exceed the theoretical maximum imposed by the PSBMA monomer cross-sectional area, confirming that the original F_SiO2_ values were underestimated; the TGA-quantified silica contents are therefore adopted in the revised manuscript. The apparent higher σ values for f_SiO2_ = 1–2 wt% in the TGA-based calculation reflect the larger relative contribution of PSBMA pyrolytic char to the 600 °C residual; these values represent lower bounds and would increase slightly upon char-yield correction.

The SI-ATRP strategy enabled successful fabrication of well-defined SiO_2_-*g*-PSBMA hybrid materials with tunable grafted chain lengths, and comprehensive characterizations validated the efficient grafting of zwitterionic PSBMA segments onto silica nanoparticle surfaces. These prepared hybrid nanoparticles lay a solid material foundation for subsequent fracturing-fluid performance evaluation under simulated Sichuan tight sandstone reservoir conditions.

### 3.2. Self-Assembly and Rheological Character of SiO_2_-g-PSBMA in Aqueous Solutions

With the well-defined molecular structures of SiO_2_-*g*-PSBMA hybrids established in the preceding section, we further probed inter-chain zwitterionic interactions, aqueous self-assembly behaviors and the corresponding rheological performances for fracturing-fluid application.

**Figure 3:**
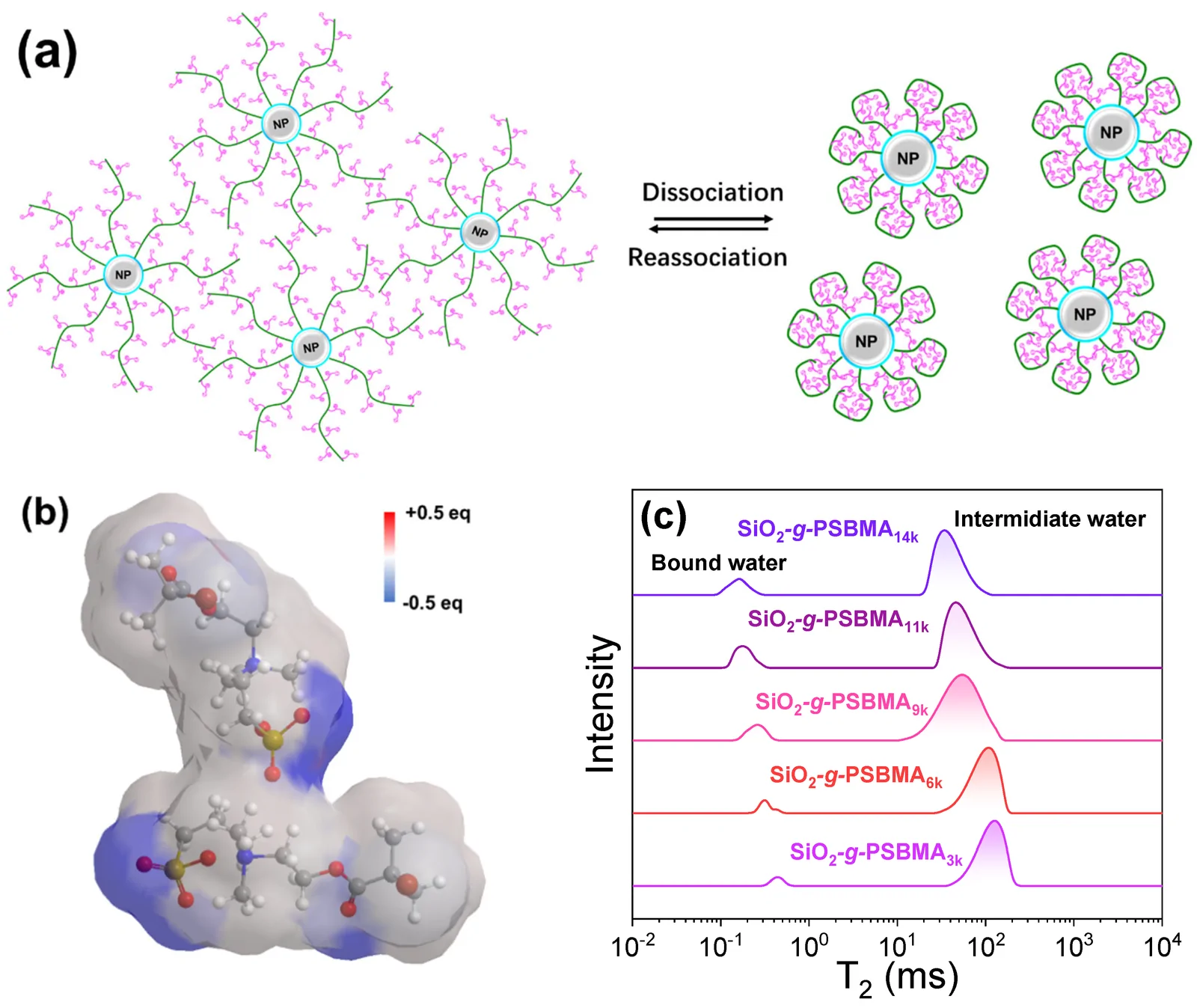

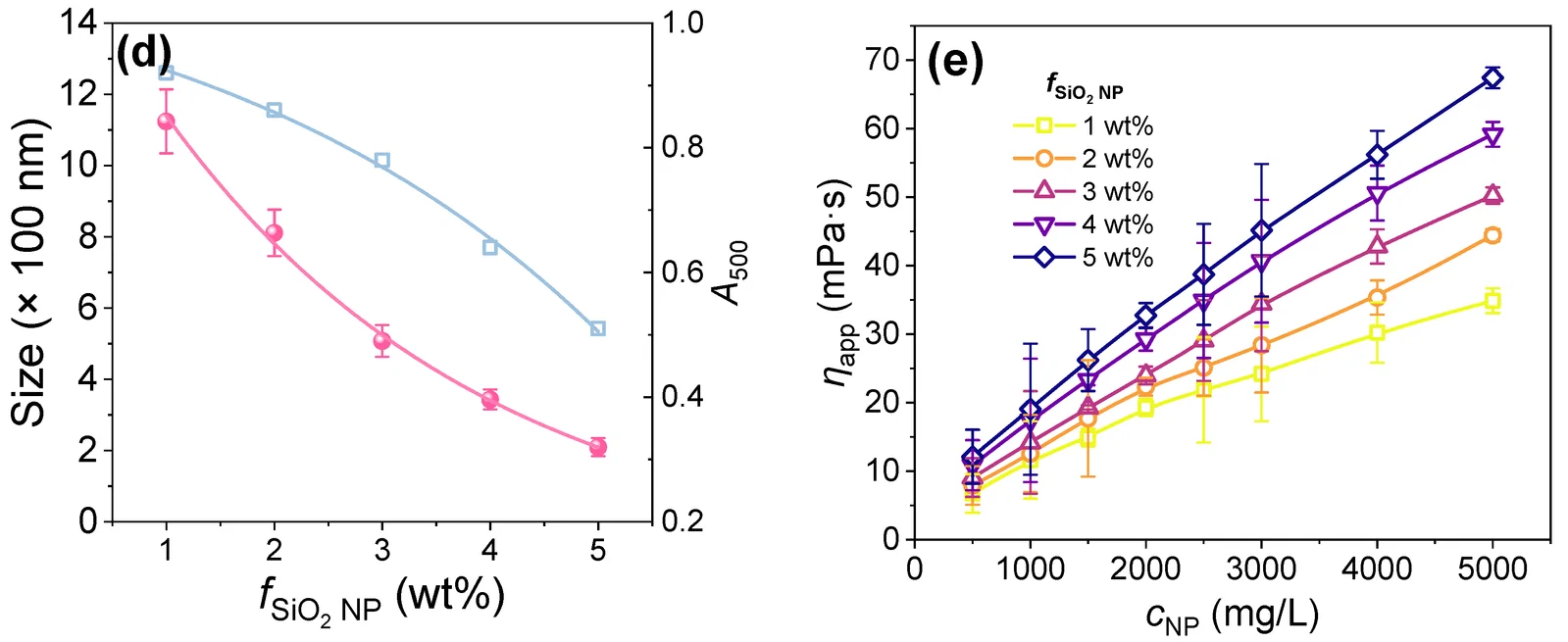
(**a**) Schematical illustration of the disassociation–reassociation process of SiO_2_-*g*-PSBMA hybrids during oscillation. (**b**) ESP distribution map of SBMA molecules, obtained by DFT analysis via NBO method. (**c**) T_2_ spectra of SiO_2_-*g*-PSBMA hybrids in aqueous solutions with the concentration of 1 mg mL^−1^. (**d**) Effects of *f*_SiO2 NP_ on size of nanoparticles and absorbance at 500 nm determined by DLS and UV-Vis analysis (1 mg mL^−1^). (**e**) Rises in *η*_app_ with increasing *c*_NP_ of SiO_2_-*g*-PSBMA hybrids at various *f*_SiO2 NP_. Data are presented as mean ± standard deviation (S.D.) with three independent samples (*n* = 3).

Figure 3a depicts the reversible dissociation–reassociation equilibrium driven by intra- and intermolecular electrostatic dipole–dipole attractions among sulfobetaine side-chains under oscillatory shear stimuli. To reveal the intrinsic electrostatic origin of such interchain interactions at the molecular level, density functional theory (DFT) simulation coupled with natural bond orbital (NBO) analysis was implemented to compute the electrostatic potential (ESP) distribution of SBMA monomer unit, as visualized in Figure 3b [51]. The ESP mapping clearly displays spatially separated charge domains: electro-positive regions are concentrated on quaternary ammonium (-N^+^(CH_3_)_2_-) moieties, while electro-negative domains are localized on sulfonate (-CH_2_-SO_3_^−^) groups. This distinct charge separation endows PSBMA grafts with strong intermolecular dipole–dipole attraction, which dominates the conformational flexibility of polymer chains tethered on the silica surface. Such zwitterionic association further cascades to govern colloidal aggregation status, hydration environment and ultimately the bulk viscous property of hybrid aqueous dispersion.

^1^H T_2_ relaxation NMR measurements were conducted at a fixed concentration of 1 mg mL^−1^ to discriminate tightly bound water and intermediate water surrounding SiO_2_-*g*-PSBMA nanoparticles, providing direct insight into polymer–water interfacial interactions (Figure 3c) [52]. Two characteristic water relaxation populations can be resolved across all samples. The short-relaxation-time component below 1 ms is assigned to bound water molecules tightly constrained by zwitterionic groups, whereas the prominent peak falling within 10–100 ms corresponds to intermediate water. With the increase in grafted PSBMA chain length, the relative peak area of bound water rises remarkably and the corresponding T_2_ value gradually shifts from 0.67 to 0.12 ms, confirming that longer zwitterionic grafted chains recruit more water molecules via dipole–dipole hydration interactions [53].

Wide-angle X-ray diffraction (WAXD, Figure S3) was used to characterize the crystallinity of pristine SiO_2_ NPs, initiator-modified SiO_2_ NPs and SiO_2_-*g*-PSBMA_14k_. Pristine silica displayed a broad amorphous halo at 2θ ≈ 22°, and no sharp crystalline peaks appeared for either the modified silica or final hybrid, confirming their fully amorphous nature. After PSBMA grafting, the low-angle baseline intensity increased and the amorphous hump slightly shifted, indicating the successful introduction of amorphous PSBMA layers; no long-range ordered packing was formed, demonstrating that zwitterionic interactions correspond to short-range dynamic associations rather than stable crystalline domains. Dynamic light scattering (DLS) and UV-Vis absorption measurements were carried out to quantify the hydrodynamic dimensions and light-scattering properties of hybrid particles under varying *f*_SiO2 NP_, as presented in Figure 3d. As *f*_SiO2 NP_ increased from 1 to 5 wt%, the hydrodynamic size decreased monotonically from 1220 nm down to 210 nm (Figure S4). Meanwhile, the absorbance at 500 nm (*A*_500_) declined correspondingly from 0.92 to 0.31 (Figure S5). Higher nano-initiator loading produces shorter PSBMA graft chains, which suppresses inter-particle zwitterionic bridging and reduces the degree of colloidal aggregation, thereby yielding smaller hydrodynamic particle dimensions and weaker light-scattering absorbance.

Figure 3e presents the evolution of *η*_app_ as a function of nanoparticle concentration *c*_NP_ for hybrid samples with five different *f*_SiO2 NP_ gradients ranging from 1 to 5 wt%. For every group, apparent viscosity exhibits positive concentration dependence: *η*_app_ continuously elevates as *c*_NP_ increases from 500 mg L^−1^ to 5000 mg L^−1^. At *c*_NP_ = 5000 mg L^−1^, the apparent viscosity increases sequentially from 34 up to 68 mPa·s. Under identical particle concentration, higher *f*_SiO2 NP_ consistently delivers larger apparent viscosity values. This viscosity discrepancy originates from the tunable transient physical network constructed by dynamic dissociation–reassociation of zwitterionic segments. Reversible inter-particle physical cross-links are formed within the aqueous medium, which significantly thickens the fluid system and meets the fundamental viscosity requirement for fracturing-fluid usage. Taken together, these multi-dimensional characterizations verify that inter-chain zwitterionic interactions of SiO_2_-*g*-PSBMA hybrids can be rationally modulated by adjusting grafted PSBMA chain length, which further precisely tunes particle self-assembly morphology, interfacial hydration condition and bulk solution rheological behaviors.

**Figure 4:**
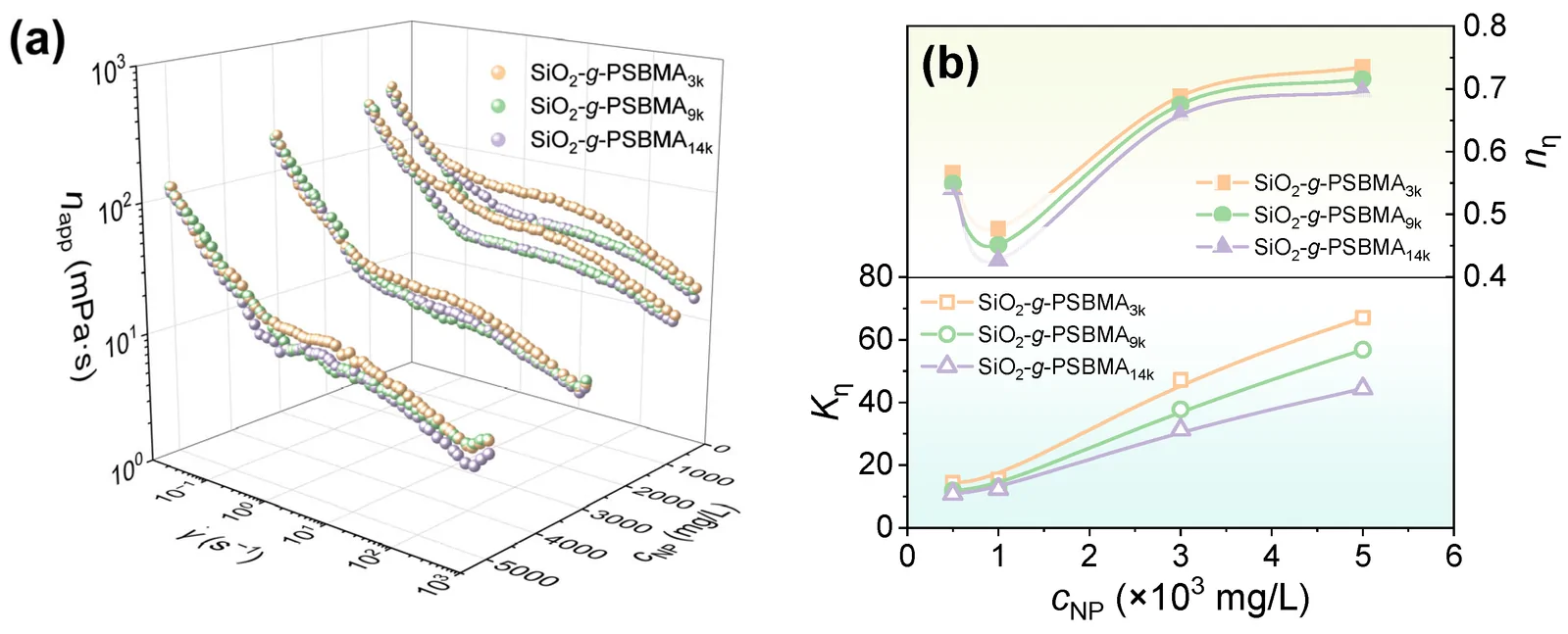

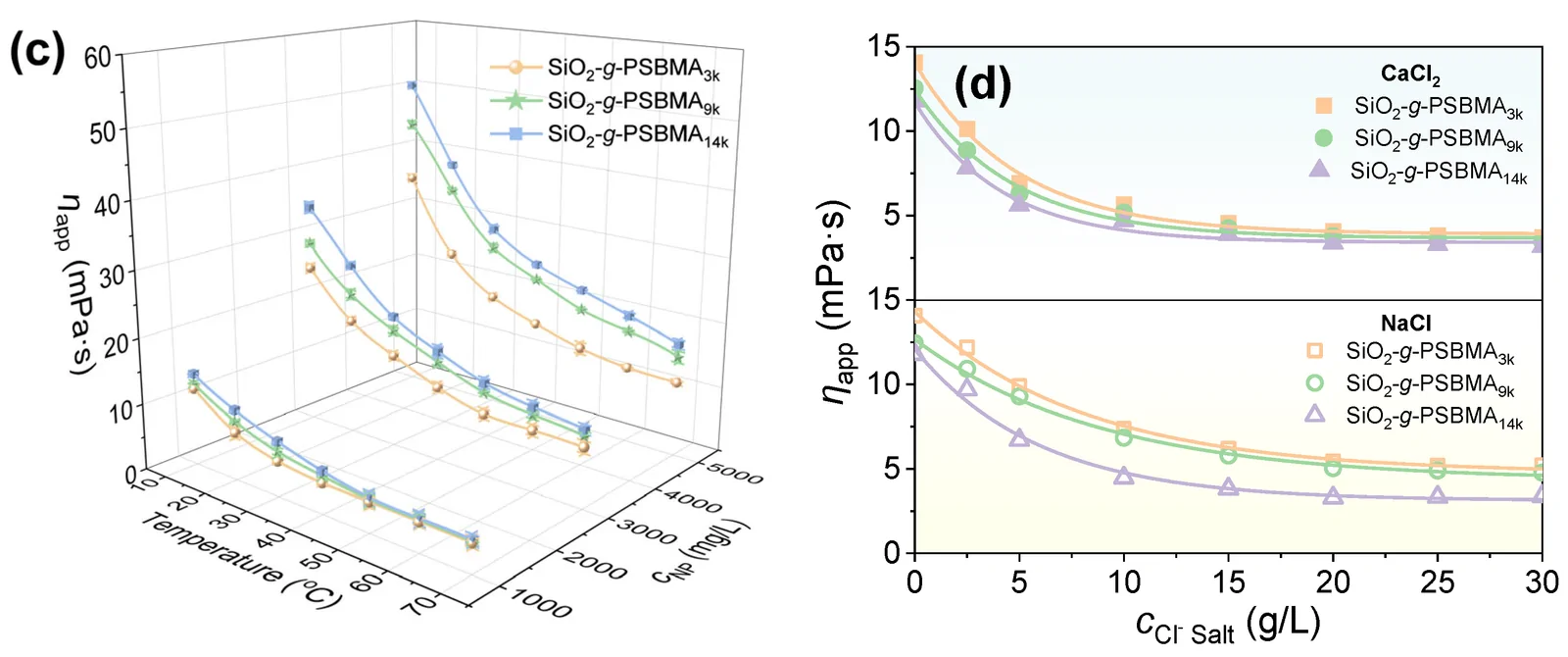
(**a**) Plots of *η*_app_ of various SiO_2_-*g*-PSBMA and $\dot{\gamma }$ with various *c*_NP_ at 30 °C. (**b**) Effects of *c*_NP_ on *K*_η_ and *n*_η_ fitted by power-law function. (**c**) Decreases in *η*_app_ of various SiO_2_-*g*-PSBMA during heating process with various *c*_NP_ at 30 °C. (**d**) Decreases in *η*_app_ of various SiO_2_-*g*-PSBMA under mineralized environments simulated by NaCl and CaCl_2_ solutions with increasing concentration (*c*_NP_ = 1 g L^−1^, 30 °C). Data are presented as mean ± standard deviation (S.D.) with three independent samples (*n* = 3).

Subsequently, under practically relevant external stimuli, including shear field, temperature and mineralized brine environments, the rheological performances of SiO_2_-*g*-PSBMA aqueous fluids were systematically evaluated, which directly determine the operational stability of fracturing fluids under real reservoir conditions. Figure 4a presents three-dimensional surface plots of *η*_app_ as a function of shear rate ($\dot{\gamma }$) and nanoparticle concentration measured at 30 °C. All hybrid formulations exhibit prominent shear-thinning non-Newtonian behavior: apparent viscosity decreases exponentially upon increasing shear rate across the full concentration range. Typically, at *c*_NP_ = 5000 mg L^−1^, *η*_app_ drops from 68 mPa·s at down to 3 mPa·s with increasing $\dot{\gamma }$ from 10^−2^ to 10^3^ s^−1^. Raising nanoparticle concentration consistently elevates the absolute viscosity at identical shear rates, owing to more abundant zwitterionic dynamic cross-links within the aqueous network. Power-law fitting was applied to extract the consistency coefficient (*K*_η_) and flow-behavior index (*n*_η_), and the fitted parameters versus nanoparticle concentration are summarized in Figure 4b. As *c*_NP_ increases from 500 to 5000 mg L^−1^, *K*_η_ increases monotonically from 12 up to 64 mPa·s, reflecting continuously enhanced thickening capability. Meanwhile, *n*_η_ maintains values below 0.7 for all samples, firmly verifying the typical pseudoplastic characteristic that facilitates convenient pumping during fracturing construction.

The temperature-dependent rheological response upon heating from 30 °C to 90 °C was investigated at varied nanoparticle loadings, as shown in Figure 4c. For each sample, *η*_app_ shows a steady decline with elevated temperature, and the thermal motion weakens the reversible zwitterionic inter-particle associations and partially disrupts the transient physical network. Even at 90 °C, the hybrid fluid still retains non-negligible *η*_app_ above 8 mPa·s at high particle loading, demonstrating acceptable thermal stability for moderate-temperature reservoir applications. Figure S6 illustrates the combined influence of $\dot{\gamma }$ and temperature on *η*_app_ for SiO_2_-*g*-PSBMA aqueous solutions. For each sample, *η*_app_ decreases simultaneously with rising $\dot{\gamma }$ and elevated temperature, where thermal agitation disrupts the dynamic zwitterionic physical networks and further lowers viscosity. Nevertheless, pseudoplastic performance persists over the investigated temperature window from 30 to 90 °C, confirming that the non-Newtonian rheological character is well-preserved under heating conditions.

To further evaluate the viscoelastic properties relevant to fracturing-fluid applications, oscillatory shear rheology measurements were performed. Frequency sweeps (0.1–100 Hz) and stress sweeps (0.1–100 Pa) were conducted at 25 °C using a parallel-plate geometry (50 mm diameter, 1 mm gap). As shown in Figure S7a, all samples exhibit a predominantly elastic response (G′ > G″) across the measured frequency range, with both moduli increasing monotonically with frequency. SiO_2_-*g*-PSBMA_14k_ displays the highest absolute moduli (G′ ≈ 15 Pa at 0.1 Hz to ≈ 300 Pa at 100 Hz; G″ ≈ 6–150 Pa), followed by SiO_2_-*g*-PSBMA_11k_ (G′ ≈ 5–80 Pa) and neat PSBMA_10k_ (G′ ≈ 0.3–30 Pa). The enhanced G′ in the grafted hybrids indicates that the SiO_2_ cores act as physical crosslinking junctions, reinforcing the elastic network. Notably, neat PSBMA_10k_ exhibits a crossover from viscous-dominated (G″ > G′) to elastic-dominated (G′ > G″) behavior at low frequencies, whereas both grafted hybrids maintain G′ > G″ even at 0.1 Hz, confirming that nanoparticle grafting extends the elastic response to lower frequencies—beneficial for proppant suspension. Stress sweeps (Figure S7b) reveal that the linear viscoelastic region extends to approximately 1 Pa for SiO_2_-*g*-PSBMA_14k_, 5 Pa for SiO_2_-*g*-PSBMA_11k_, and 10 Pa for PSBMA_10k_, beyond which G′ decreases (first stress zone), marking the onset of network breakdown.

The observed shear-thinning behavior and viscosity range (34–68 mPa·s at 5000 mg L^−1^) are consistent with recent reports on zwitterionic polymer-grafted nanoparticle systems for reservoir applications. Si et al. demonstrated that a salt-responsive zwitterionic polymer (PTAD) exhibited enhanced viscosity in high-salinity brines, with Ca^2+^ increasing viscosity by 70.21% at 4000 mg L^−1^, and achieved a 30.3% improvement in oil recovery ratio over conventional PAM in core flooding [21]. Similarly, Li et al. reported that dendritic mesoporous silica nanoparticles reinforced a betaine amphiphilic polymer system, improving viscosity, salt resistance, and thermal tolerance, with tertiary oil recovery reaching 17.91%—nearly double that of the conventional SiO_2_/polymer system (9.48%) [54].

The salt-tolerance performances with NaCl and CaCl_2_ were further characterized to illustrate the rheological stability of SiO_2_-*g*-PSBMA aqueous solutions under simulated mineralized conditions (Figure 4d). Progressive addition of inorganic salt leads to continuous viscosity decay for all SiO_2_-*g*-PSBMA systems, as salt ions might screen the electrostatic dipole–dipole interactions between zwitterionic side groups. Divalent Ca^2+^ induces more severe viscosity deterioration than monovalent Na^+^ under equivalent mass concentration. At salt concentration of 30 g L^−1^, the apparent viscosity falls to 3.2 mPa·s in CaCl_2_ solution versus 5.1 mPa·s in NaCl medium. Despite the viscosity loss in high-salinity surroundings, the zwitterionic hybrid system still maintains residual effective viscosity, confirming its reasonable salt-resistance capacity. Collectively, these rheological results demonstrate that SiO_2_-*g*-PSBMA fluids possess favorable shear-thinning behavior, qualified thermal tolerance and moderate salt-resistance capacity, which satisfy core performance requirements for fracturing-fluid exploitation within complex subsurface environments. Figure S8 presents three-dimensional rheological surfaces describing the mutual dependence of *η*_app_, $\dot{\gamma }$ and NaCl salinity for SiO_2_-*g*-PSBMA samples at a fixed particle concentration of 1 g L^−1^ and 30 °C. All hybrid fluids retain typical shear-thinning behavior across the whole NaCl concentration range, while increasing sodium chloride concentration systematically suppresses the absolute viscosity values due to electrostatic screening of zwitterionic dipole–dipole interactions. Even under high-salinity conditions, obvious shear-thinning characteristics can still be observed, demonstrating that the hybrid system maintains its non-Newtonian flow feature in saline environments.

Systematic interfacial and adsorption measurements uncovered the temperature- and chain-length-dependent interfacial activity and rock-adsorption behavior of SiO_2_-*g*-PSBMA, demonstrating that longer PSBMA graft chains favored interfacial assembly and higher adsorption capacity on the sandstone matrix. Even under high-salinity brine environments mimicking formation water, the zwitterionic hybrids retained prominent interfacial tension reduction capability.

### 3.3. Fracturing Fluid Based SiO_2_-g-PSBMA Hybrids for Highly Efficient Recovery

**Figure 5:**
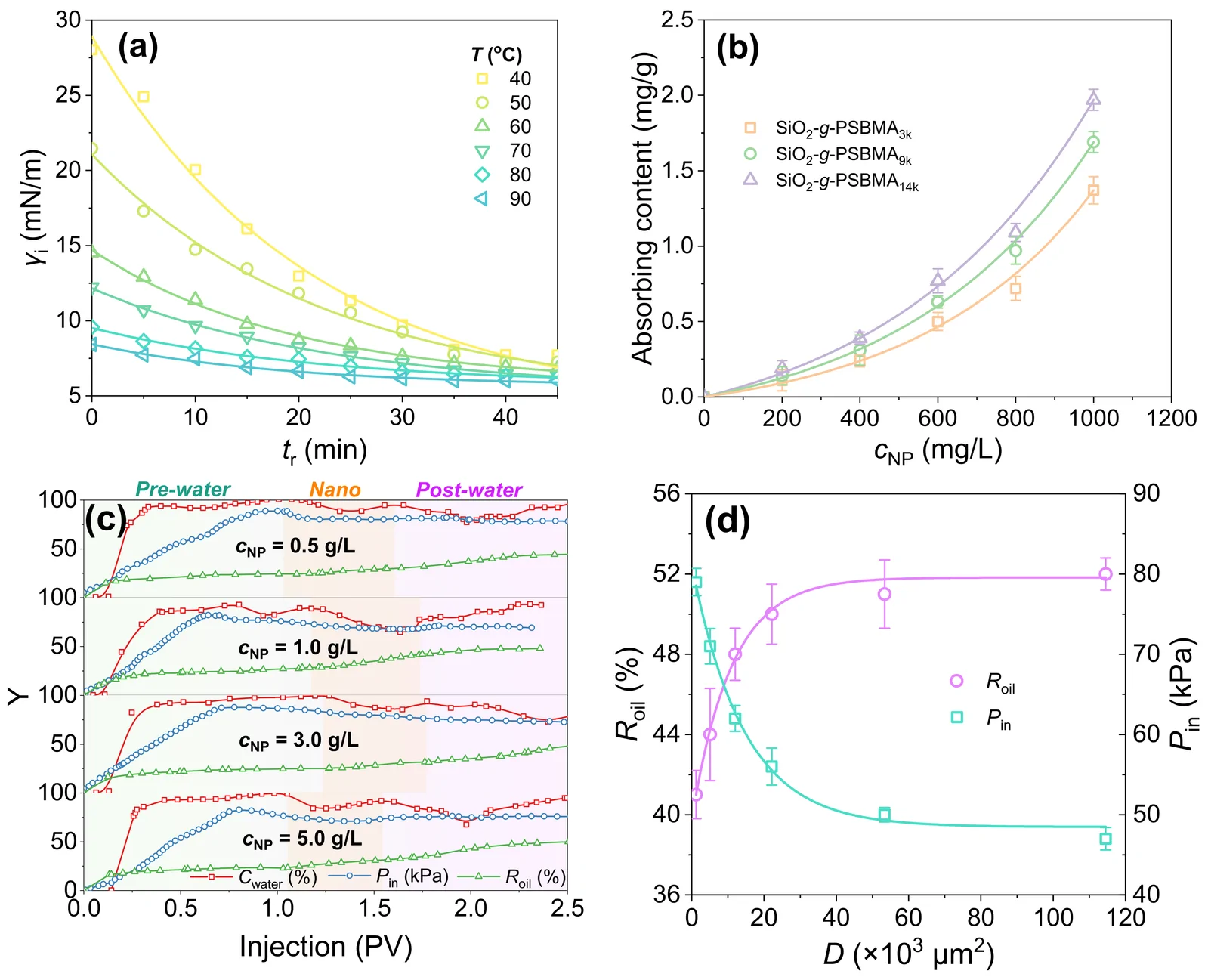
(**a**) Decreases in interface tension (*γ*_i_) on the interface between SiO_2_-*g*-PSBMA_14k_ solutions (1 g L^−1^) and core samples with prolonging recovering time (*t*_r_) at various temperatures. (**b**) Relationships between absorbing contents of various SiO_2_-*g*-PSBMA solutions on dry core samples with various *c*_NP_ at 90 °C (permeability, *D =* 52 × 10^3^ μm^2^; flow velocity, *V*_in_ = 0.2 mL min^−1^). (**c**) Changes in water content (*C*_water_), injection pressure (*P*_in_) and recovery rate (*R*_oil_) of SiO_2_-*g*-PSBMA_14k_ fracturing fluid with various *c*_NP_ at 90 °C (permeability, *D =* 52 × 10^3^ μm^2^; flow velocity, *V*_in_ = 0.2 mL min^−1^). (**d**) Effects of *D* on *P*_in_ and *R*_oil_ by using SiO_2_-*g*-PSBMA_14k_ fracturing fluid at 90 °C (*c*_NP_ = 1.0 g L^−1^, *V*_in_ = 0.2 mL min^−1^). Data are presented as mean ± standard deviation (S.D.) with three independent samples (*n* = 3).

The interfacial chemical behaviors between rock cores and SiO_2_-*g*-PSBMA fluid were subsequently investigated, in which the core is mined in the Sichuan Xujiahe tight sandstone reservoir. To reveal the intrinsic mechanisms for oil displacement and pressure response during fracturing-fluid operations, Figure 5a displays the dynamic interfacial tension (*γ*_i_) evolution between the SiO_2_-*g*-PSBMA_14k_ aqueous solution (*c*_NP_ = 1.0 g L^−1^) and core-produced crude oil as a function of contact time at different temperatures ranging from 40 to 90 °C. At 40 °C, the initial interfacial tension starts at 28 mN m^−1^ and gradually falls and remains at 7.2 mN m^−1^ after 45 min contact. The *γ*_i_ value declines from 11.5 mN m^−1^ down to 5.1 mN m^−1^ and is stabilized within the same time window at 90 °C, demonstrated that the elevated temperature accelerates the interfacial-tension reduction process. All tested groups achieve obvious interfacial-tension reduction, confirming that the zwitterionic hybrid nanoparticles can efficiently assemble at the oil–water interface. Higher temperature promotes interfacial molecular rearrangement and yields lower equilibrium *γ*_i_. Complementarily, Figure S9 further evaluates the time-dependent *γ*_i_ profiles under high-NaCl-salinity environments at 90 °C. For all NaCl mass fractions spanning 10~20 wt%, *γ*_i_ decays rapidly within the initial 20 min and then levels off toward equilibrium values. Notably, lower NaCl loading gives more prominent interfacial-activity performance; the NaCl solutions with concentrations of 10 wt% system reach an equilibrium interfacial tension of 5.3 mN m^−1^, whereas the NaCl solutions with concentrations of 20 wt% stabilize at approximately 7.8 mN m^−1^. Although high-concentration NaCl weakens interfacial assembly via electrostatic screening, the SiO_2_-*g*-PSBMA_14k_ system still retains considerable interfacial-tension-reduction capacity under extreme-salinity conditions relevant to Sichuan formation brine.

Figure 5b quantifies the rock-adsorption capacity of various SiO_2_-*g*-PSBMA hybrids on dry tight sandstone core solids at 90 °C. For all samples, the adsorbed mass increases monotonically with rising nanoparticle concentration (*c*_NP_). As *c*_NP_ increases from 0 to 1000 mg L^−1^, the adsorption amount of SiO_2_-*g*-PSBMA_14k_ rises continuously from 0 up to 2.1 mg g^−1^, which is distinctly higher than SiO_2_-*g*-PSBMA_9k_ (1.7 mg g^−1^) and SiO_2_-*g*-PSBMA_3k_ (1.3 mg g^−1^) under identical loading. Longer zwitterionic PSBMA graft chains enhance rock-surface affinity, thus generating larger adsorption capacity on the sandstone matrix. Figure S10 characterizes adsorption kinetic curves at fixed (*c*_NP_ = 1.0 g L^−1^), demonstrating that adsorption content rises sharply within the first 40 min and gradually approaches saturation. The SiO_2_-*g*-PSBMA_14k_ fracture fluid exhibits a relatively slow initial adsorption rate yet the highest saturated adsorption capacity of 2.1 mg g^−1^, while short-chain SiO_2_-*g*-PSBMA_3k_ achieves adsorption saturation much faster but only reaches 1.4 mg g^−1^ at equilibrium. This kinetic difference indicates that longer grafted zwitterionic chains require more time to rearrange and anchor onto sandstone surfaces yet deliver greater ultimate surface coverage. The saturated adsorption capacity of 2.1 mg g^−1^ for SiO_2_-*g*-PSBMA_14k_ on tight sandstone is comparable to values reported for polymer-coated silica nanofluids in recent studies. Fan et al. showed that a zwitterionic surfactant-modified SiO_2_ nanofluid (CAPHS-APTES-SNP) exhibited enhanced stability and interfacial performance under high-salinity and high-temperature conditions, effectively altering rock wettability and promoting imbibition oil recovery in low-permeability reservoirs [55]. Xin et al. further demonstrated that surface-modified nano-SiO_2_ emulsions achieved 18.83% enhanced oil recovery in low-permeability sandstone via optimized segmented injection [56].

Following the standard displacement sequence including pre-water flooding, fluid flooding and post-water flooding, the multi-parameter core-flooding curves were recorded acquired on Sichuan Xujiahe-formation tight sandstone plugs at 90 °C, shown in Figure 5c. During the pre-water-flooding stage, the injection pressure builds up and oil recovery reaches a baseline value. Once SiO_2_-*g*-PSBMA_14k_ fluid is injected, both injection pressure (*P*_in_) and oil recovery (*R*_oil_) increase remarkably; higher (*c*_NP_) produces larger injection pressure and improved ultimate oil recovery. When switching to post-water flooding, the system maintains stable oil-recovery plateau without sharp drop. At *c*_NP_ of 1.0 g L^−1^, the ultimate oil recovery achieves the maximum value among these groups, demonstrating that appropriately increased nanoparticle dosage strengthens the flow-diversion effect within pore channels of tight sandstone. Figure S11 reveals the influence of injection velocity (*V*_in_) on flooding responses under constant *c*_NP_ of 1.0 g L^−1^. Across *V*_in_ of 0.05, 0.10 and 0.20 mL min^−1^, all runs reproduce the three stages including pre-water, nano and post-water flooding profile. Elevated injection velocity brings about higher injection-pressure amplitude, and slower *V*_in_ of 0.05 mL min^−1^ provides sufficient contact time between zwitterionic nanoparticles and rock pores, yielding improved ultimate oil-recovery performance. This observation suggests that low-to-moderate injection rates are favorable for fluid displacement in low-permeability tight sandstone reservoirs.

Figure 5d further illustrates how core permeability (*D*) influences *P*_in_ and *R*_oil_ for SiO_2_-*g*-PSBMA_14k_ fluid with *c*_NP_ of 1.0 g L^−1^ at 90 °C. As the average *D* increases from 5.1 × 10^3^ μm^2^ to 112.5 × 10^3^ μm^2^, *P*_in_ decreases steeply from 82 down to 49 kPa, while oil-recovery *R*_oil_ rises rapidly from 41% and gradually plateaus around 52%. Smaller pore-throat size corresponds to larger flow resistance and higher injection pressure. Meanwhile, the fluid shows prominent enhancement on oil recovery for low-permeability tight cores, which highlights its application potential for Sichuan low-permeability gas-bearing sandstone reservoirs.

It should be noted that all rheological, interfacial, and core-flooding experiments in this work were conducted at temperatures up to 90 °C. Although the target Xujiahe Formation reservoir exhibits temperatures above 120 °C, the current study establishes the structure–performance relationship of SiO_2_-*g*-PSBMA hybrids under the tested temperature range (30–90 °C) and high-salinity conditions (up to 20 wt% NaCl). Further evaluation at temperatures ≥120 °C, including high-pressure rheology and core flooding, is required before field applicability at reservoir temperature can be fully established. Core-flooding results showing improved oil recovery at moderate nanoparticle loading align with the optimal concentration range reported in recent nanofluid EOR investigations. Corredor et al. reported that a SiO_2_/PAM nanohybrid exhibited improved flow behavior through porous media and enhanced displacement performance, with experimental and numerical simulation confirming the role of nanoparticle–polymer interactions in pore-scale flow diversion [57]. Rezvani et al. demonstrated that covalently epoxysilane-grafted silica nanoparticles blended with zwitterionic surfactants improved emulsion stability and EOR performance in carbonate rocks, highlighting the benefit of covalent surface modification over physical blending [58].

Core-flooding experiments based on Xujiahe tight sandstone core plugs illustrated that nanoparticle concentration, injection velocity and pore-throat dimension collectively governed the injection pressure and oil-displacement efficiency of SiO_2_-*g*-PSBMA_14k_ fracturing fluid. The fluid achieved favorable flow diversion and oil recovery improvement in low-permeability cores, confirming its potential for further evaluation for Sichuan tight gas reservoirs. In addition, the results presented in this work are based on laboratory-scale core-flooding experiments using outcrop cores and simulated reservoir conditions (up to 90 °C). These findings establish the fundamental structure–performance relationship of SiO_2_-*g*-PSBMA hybrids and demonstrate their potential as fracturing-fluid additives under controlled laboratory conditions. Field-scale validation, including compatibility with actual formation brines and crude oils, long-term core damage evaluation, and pilot-scale testing, is required before practical deployment can be recommended.

## 4. Conclusions

In this study, structurally well-defined zwitterionic SiO_2_-*g*-poly(sulfobetaine methacrylate) (SiO_2_-*g*-PSBMA) inorganic–organic hybrid nanoparticles with controllable graft chain lengths were successfully synthesized via surface-initiated atom-transfer radical polymerization (SI-ATRP) at 80 °C. By systematically varying the silica nano-initiator feeding fraction (f_SiO2 NP_) from 1 to 5 wt%, the number-average molecular weight (M_n_) of the grafted PSBMA chains was precisely tuned from 14.2 down to 3.1 kg mol^−1^ with narrow dispersity (Đ = 1.64–1.78), and the corresponding grafting density, calculated by combining TGA, GPC, and BET analyses, ranged from 0.12 to 0.45 chains nm^−2^. Thermogravimetric analysis confirmed a two-step thermal decomposition profile for all hybrids, with residual inorganic fractions at 600 °C increasing monotonically from 7% to 73% as f_SiO2 NP_ increased from 1 to 5 wt%.

Rheological evaluation revealed that the SiO_2_-*g*-PSBMA hybrids exhibited pronounced shear-thinning behavior with apparent viscosities of 34–68 mPa·s at 5000 mg L^−1^, and retained viscosities above 8 mPa·s at 90 °C under high particle loading. Interfacial tension measurements demonstrated that the long-chain SiO_2_-*g*-PSBMA_14k_ fluid reduced the oil–water interfacial tension from 28 to 5.1 mN m^−1^ at 90 °C and maintained effective tension reduction (7.8 mN m^−1^) even at 20 wt% NaCl. Rock-adsorption studies on tight sandstone showed that longer graft chains achieved a higher saturated adsorption capacity of 2.1 mg g^−1^, attributed to the enhanced multidentate contact and steric stabilization afforded by extended PSBMA brushes. Core-flooding experiments using natural Xujiahe tight sandstone cores at 90 °C demonstrated that the optimal SiO_2_-*g*-PSBMA_14k_ concentration of 1.0 g L^−1^ achieved ultimate oil recovery (R_oil_) of approximately 41% for low-permeability cores (*D* = 5.1 × 10^3^ μm^2^) and approximately 52% for higher-permeability cores (*D* = 112.5 × 10^3^ μm^2^).

It should be noted that all experiments were conducted within the temperature range of 30–90 °C and under laboratory-scale conditions. Although the target Xujiahe Formation reservoir exhibits temperatures above 120 °C, the current work establishes the chain-length-dependent structure–performance relationship of SiO_2_-*g*-PSBMA hybrids under the tested temperature range and high-salinity conditions (up to 20 wt% NaCl). Further evaluation at temperatures ≥120 °C, including high-pressure rheology, long-term thermal aging, and field-scale core flooding, is required before practical deployment can be recommended.

## Figures and Tables

**Table 1:** Grafting density of SiO_2_-*g*-PSBMA hybrids determined by TGA–GPC and FSiO_2_–GPC combined analyses.

*f*_SiO_2_ NP_ (wt%)	*M*_n_ (kg mol^−1^)	TGA Method			F_SiO_2__ Method		
		*w*_SiO2_ (%)	*N*_chains_ (per NP)	σ (nm^−2^)	*F*_SiO2 NP_ (%)	*N*_chains_ (per NP)	σ (nm^−2^)
1	14.2	7.0	5192	4.13	1.80	21,321	16.97
2	11.4	13.0	3258	2.59	2.63	18,058	14.37
3	8.6	45.0	789	0.63	3.45	18,059	14.37
4	5.9	62.0	576	0.46	4.28	21,061	16.76
5	3.1	73.0	662	0.53	5.10	33,311	26.51

Note: M_n_ was measured by GPC (HFIP, PMMA standards) after TBAF cleavage. w_SiO2_ for the TGA method was obtained from the 600 °C residual; F_SiO2_ for the Figure 2d method was taken from the original Figure 2d (interpolated for f_SiO2_ = 2–4 wt%). The TGA-based σ values for f_SiO2_ = 1–2 wt% are lower-bound estimates due to PSBMA char contribution. The F_SiO2_-based σ values exceed the theoretical maximum (~2–3 chains nm^−2^), confirming systematic underestimation of silica content in the original Figure 2d.

## Data Availability

The original contributions presented in this study are included in the article. Further inquiries can be directed to the corresponding author.

## Supplementary Materials

The following supporting information can be downloaded at: https://www.mdpi.com/article/10.3390/polym18202497/s1.

## References

[1] Striolo A., Cole D.R. (2017). Understanding Shale Gas: Recent Progress and Remaining Challenges. Energy Fuels.

[2] Sun C., Nie H., Dang W., Chen Q., Zhang G., Li W., Lu Z. (2021). Shale Gas Exploration and Development in China: Current Status, Geological Challenges, and Future Directions. Energy Fuels.

[3] You L., Zhang N., Kang Y., Xu J., Cheng Q., Zhou Y. (2021). Zero Flowback Rate of Hydraulic Fracturing Fluid in Shale Gas Reservoirs: Concept, Feasibility, and Significance. Energy Fuels.

[4] Da Q., Yao C., Zhang X., Li L., Lei G. (2024). Reservoir Damage Induced by Water-Based Fracturing Fluids in Tight Reservoirs: A Review of Formation Mechanisms and Treatment Methods. Energy Fuels.

[5] Yang J., Dong T., Yi J., Jiang G. (2024). Development of Multiple Crosslinked Polymers and Its Application in Synthetic-Based Drilling Fluids. Gels.

[6] Li S., Long Y., Chen C., Ren H., Chang Y., Zhuo S., Yin N., Luo C., Lu W. (2025). Integrated Fracturing Strategy for Deep Tight Sandstone Gas Reservoirs in the Xujiahe Formation, Western Sichuan. Sci. Rep..

[7] Han J., Sun J., Lv K., Yang J., Li Y. (2022). Polymer Gels Used in Oil–Gas Drilling and Production Engineering. Gels.

[8] Luo M., Si X., Li M., Huang Y., Li Q., Li C. (2022). Experimental Study on the Temporary Plugging Performance of Magnetic Responsive Hydrogel in Hydraulic Fracturing of Hydrocarbon Reservoirs. Colloids Surf. A.

[9] Zhong C., Chen R., Liu B., Pu S., Hou D. (2024). Trends in Polyacrylamide Utilization and Treatment for Hydraulic Fracturing. npj Mater. Sustain..

[10] Al-Majali M.R., Zhang M., Al-Majali Y.T., Trembly J.P. (2025). Impact of raw material on thermo-physical properties of carbon foam. Can. J. Chem. Eng..

[11] Davoodi S., Al-Shargabi M., Wood D.A., Rukavishnikov V.S., Minaev K.M. (2024). Synthetic Polymers: A Review of Applications in Drilling Fluids. Pet. Sci..

[12] Yang S., Yu W., Zhao M., Ding F., Zhang Y. (2024). A Review of Weak Gel Fracturing Fluids for Deep Shale Gas Reservoirs. Gels.

[13] Ahmad H.M., Iqbal T., Kamal M.S., Al-Harthi M.A. (2020). Influence of Hydrophobically Modified Polymer and Titania Nanoparticles on Shale Hydration and Swelling Properties. Energy Fuels.

[14] Al-Mohammed A.M., Nasr-El-Din H.A., Al-Aamri A.D., Al-Fuwaires O.A. (2007). Degradation of High-pH Borate Fracturing Gels Made from Guar Gum and Hydroxypropyl Guar. Proceedings of the International Petroleum Technology Conference (IPTC 2007), Dubai, United Arab Emirates, 4–6 December 2007.

[15] de Leon A.C.C., da Silva Í.G.M., Pangilinan K.D., Chen Q., Caldona E.B., Advincula R.C. (2021). High Performance Polymers for Oil and Gas Applications. React. Funct. Polym..

[16] Liu X., Liu K., Gou S., Liang L., Luo C., Guo Q. (2014). Water-Soluble Acrylamide Sulfonate Copolymer for Inhibiting Shale Hydration. Ind. Eng. Chem. Res..

[17] Bagaria H.G., Yoon K.Y., Neilson B.M., Cheng V., Lee J.H., Worthen A.J., Xue Z., Huh C., Bryant S.L., Bielawski C.W. (2013). Stabilization of Iron Oxide Nanoparticles in High Sodium and Calcium Brine at High Temperatures with Adsorbed Sulfonated Copolymers. Langmuir.

[18] Wang J., Ran D., Lu H., Zhang J., Wu Y. (2024). Realizing the Efficient Dissolution of Drag Reducer upon pH-Induced Demulsification of Inverse Polymer Emulsion. Langmuir.

[19] Lei S., Sun J., Lv K., Zhang Q., Yang J. (2022). Types and Performances of Polymer Gels for Oil-Gas Drilling and Production: A Review. Gels.

[20] Abdel-Azeim S. (2021). Phase Behavior and Interfacial Properties of Salt-Tolerant Polymers: Insights from Molecular Dynamics Simulations. ACS Appl. Polym. Mater..

[21] Si S., Liu Y., Dai C., Zou C., Zhuo L., Li X., Yang N., Ma D. (2025). Salt Resistance Mechanism and Oil Displacement Performance Evaluation of a New Zwitterionic Polymer. Energy Fuels.

[22] Wang S., Sun Z., Zhu Q., Cao X., Feng Y., Yin H. (2024). In Situ Generated Hydrogels Exhibiting Simultaneous High-Temperature and High-Salinity Resistance for Deep Hydrocarbon Reservoir Exploitation. Ind. Eng. Chem. Res..

[23] Chen Y., Li J., Song Q., Bu C., Ye K., He F., Lv Z., Chen X. (2024). Research on Flow Patterns of Two-Phase Fracturing Fluid in Hydraulic Fracture Considering the Fluid Leak-off. ACS Omega.

[24] Huang C., Mu L., Gong X. (2025). Development and Characterization of Environmentally Responsive Thickening Agents for Fracturing Fluids in Shale Gas Reservoir Stimulation. Processes.

[25] Yu Y., Zhang H., Xu P., Zhang X., Wang H., Hu M., Guo J. (2025). Effect of Ultra-High Temperature Degradation on the Physical Properties and Chemical Structure of an AMPS-Based Copolymer Oil-Well Cement Additive PADIM in Aqueous Solution. Polymers.

[26] Srivastava A., Mandal P., Kumar R. (2016). Solid State Thermal Degradation Behaviour of Graft Copolymers of Carboxymethyl Cellulose with Vinyl Monomers. Int. J. Biol. Macromol..

[27] Liu X., Wang A., Wang C., Qu J., Wen Y., Chen B., Wang Z., Wu B., Yuan Z., Wei B. (2019). Preparation and Performance of Salt Tolerance and Thermal Stability Cellulose Nanofibril Hydrogels and Their Application in Drilling Engineering. Pap. Biomater..

[28] Wang S., Xiong P., Liu X., Meng F., Ma Q., Song C., Zhang Q., Che Y. (2022). A Hydroxypropyl Methyl Cellulose-Based Graft Copolymer with Excellent Thermothickening and Anti-salt Ability for Enhanced Oil Recovery. Energy Fuels.

[29] Balding P., Li M., Wu Q., Volkovinsky R., Russo P. (2020). Cellulose Nanocrystal–Polyelectrolyte Hybrids for Bentonite Water-Based Drilling Fluids. ACS Appl. Bio Mater..

[30] Zhao J., Zhang M., Wang C., Yu W., Zhu Y., Zhu P. (2025). First-principles study of CO, NH3, HCN, CNCl, and Cl2 gas adsorption behaviors of metal and cyclic C–metal B-and N-site-doped h-BNs. Electron. Mater. Lett..

[31] Bahamdan W.H., Daly W.H. (2006). Poly(oxyalkylene) Grafts to Guar Gum with Applications in Hydraulic Fracturing Fluids. Polym. Adv. Technol..

[32] Jing B., Zhu Y., Zhao W., Jiang W., Zhang S., Huang B., Du G. (2025). A Zwitterionic Copolymer at High Temperature and High Salinity for Oilfield Fracturing Fluids. Polymers.

[33] Gao S., Lin D., Li A., Deng L., Dong A., Zhang J. (2025). A Zwitterionic Polymer Filtrate Reducer with Enhanced Anti-Polyelectrolyte Effect for High-Temperature and High-Salinity Conditions. Chem. Eng. J..

[34] Fevola M.J., Kellum M.G., Hester R.D., McCormick C.L. (2004). pH-Responsive Ampholytic Terpolymers of Acrylamide, Sodium 3-Acrylamido-3-methylbutanoate, and (3-Acrylamidopropyl)trimethylammonium Chloride. II. Solution Properties. J. Polym. Sci. Part A Polym. Chem..

[35] Matyjaszewski K., Xia J. (2001). Atom Transfer Radical Polymerization. Chem. Rev..

[36] Matyjaszewski K. (2018). Advanced Materials by Atom Transfer Radical Polymerization. Adv. Mater..

[37] Lego B., Francois M., Skene W.G., Giasson S. (2009). Polymer Brush Covalently Attached to OH-Functionalized Mica Surface via Surface-Initiated ATRP: Control of Grafting Density and Polymer Chain Length. Langmuir.

[38] Liu Y.F., Li W., Xiao Z.H., Ji S.W., Liu Q., Tang Y.F., Zhang Y., Wang J.M., Zhang Y., Wang J.M. (2026). Recent Progress in Organic Inhibitors for Anticorrosion in Complex Acid Environments. Coatings.

[39] Cheraghian G., Hendraningrat S. (2016). A Review of Applications of Nanotechnology in the Enhanced Oil Recovery. Adv. Colloid Interface Sci..

[40] Hendraningrat S., Li S., Torsaeter O. (2013). A Coreflood Investigation of Nanofluid Enhanced Oil Recovery in Low-Permeability Berea Sandstone. Int. J. Nano Dimens..

[41] Hendraningrat S., Torsaeter O. Metal Oxide-Based Nanoparticles: Revealing Their Interaction with Sandstones in Aqueous Media for Enhanced Oil Recovery. Proceedings of the SPE Europec Featured at 78th EAGE Conference and Exhibition.

[42] Maghzi A., Mohammadi S., Ghazanfari M.H., Kharrat R., Masihi M. (2014). Monitoring Wettability Alteration by Silica Nanoparticles during Water Flooding to Heavy Oil in a Five-Spot System: A Pore-Level Investigation. Exp. Therm. Fluid Sci..

[43] Zargartalebi M., Barati N., Kharrat R. (2014). Enhancement of Surfactant Flooding Performance by the Use of Silica Nanoparticles. Fuel.

[44] Al-Anssari S., Arif M., Wang S., Barifcani A., Lebedev M., Iglauer S. (2018). Wettability Alteration of Oil-Wet Carbonate by Silica Nanofluid. J. Colloid Interface Sci..

[45] Alsaba M.T., Al Dushaishi M.F., Abbas A.H., Alsaba A.M., Almalik M.S., Al-Rubaii B.A. (2020). A Comprehensive Review of Nanoparticles Applications in the Oil and Gas Industry. J. Pet. Explor. Prod. Technol..

[46] Yousefvand H., Jafari A. (2015). Aluminum Oxide Nanoparticles for Enhanced Oil Recovery. Int. J. Nano Dimens..

[47] Bayat A.E., Junin R., Shamshirband S., Chong W.T. (2015). Application of Al_2_O_3_ Nanoparticles for Enhancing Thermal Stability of Xanthan Gum Solutions for Efficient Hydraulic Fracturing. J. Nat. Gas Sci. Eng..

[48] Ogolo N.A., Olafuyi O.A., Onyekonwu M.O. Enhanced Oil Recovery Using Nanoparticles. Proceedings of the SPE Saudi Arabia Section Technical Symposium and Exhibition.

[49] Zhang K., Liu X.F., Wang D.B., Zheng B., Chen T.H., Wang Q., Bai H., Yao E.D., Zhou F.J. (2024). A Review of Reservoir Damage during Hydraulic Fracturing of Deep and Ultra-Deep Reservoirs. Pet. Sci..

[50] Lu T., Chen F. (2012). Multiwfn: A Multifunctional Wavefunction Analyzer. J. Comput. Chem..

[51] Liu Y.F., Zhou L., Wan X.C., Tang Y.F., Liu Q., Li W., Liao J.B. (2024). Synthesis and Characterization of a Temperature-Sensitive Microcapsule Gelling Agent for High-Temperature Acid Release. ACS Omega.

[52] Li A., Li J.-L., Zhang J.-M., Ma J.-Y., Wu Y.-X. (2025). In-situ Synthesis of Chemically Stable Hybrid Networks of Poly(thioctic acid) with Fe^3+^ via Controlled/Living Cationic Ring-Opening Polymerization. Macromol. Rapid Commun..

[53] Li A., Wang Y., Zhu H., Wu Y.-X. (2026). Hydrophilic Antifouling Poly(thioctic acid)–Fe^3+^ Hybrid Films via Hydration. J. Mater. Chem. A.

[54] Li D., Wang Y., Bai B., Xu N., Shi W., Zhang Y., Ding W., Ma P., Gao Z. (2025). Reinforcing Polymer Flooding System with Dendritic Mesoporous Silica Nanoparticles for Improved Oil Recovery. Energy Fuels.

[55] Fan Q., Bai Y., Wang K., Zhan Y., Luo M. (2025). Performance of Imbibition Oil Recovery Enhanced by a Novel Chemically Modified SiO_2_ Nanofluid for Low-Permeability Reservoirs. ACS Omega.

[56] Xin M., Zhang D., Zhang Y., Chen J., Yan L., Qin Y., Zhang Q. (2025). Optimization of Flooding Parameters and Enhanced-Efficiency Development for Surface-Modified Nano-SiO_2_ Emulsion in Low-Permeability Sandstone Reservoirs. PLoS ONE.

[57] Corredor L.M., Espinosa C., Delgadillo C.L., Llanos S., Castro R.H., Quintero H.I., Ruiz Cañas M.C., Romero Bohorquez A.R., Manrique E. (2024). Flow Behavior through Porous Media and Displacement Performance of a SILICA/PAM Nanohybrid: Experimental and Numerical Simulation Study. ACS Omega.

[58] Rezvani H., Binks B.P., Nguyen D. (2024). Surfactant-Nanoparticle Formulations for Enhanced Oil Recovery in Calcite-Rich Rocks. Langmuir.

