# Supplementary material for: SiO2-g-Poly(sulfobetaine methacrylate) Inorganic–Organic Hybrids as Fracturing Fluids Under Harsh Reservoir Conditions
Source: Polymers (Basel). 2026 Oct 12;18(20):2497. doi: 10.3390/polym18202497 (PMC13708987; doi:10.3390/polym18202497)
Supplement: Supplementary file 1 [file polymers-18-02497-s001.zip › polymers-4571689-supplementary.pdf]

Supporting Information

SiO_2-*g*-Poly(sulfobetaine methacrylate) Inorganic-Organic Hybrids as Fracturing Fluids under Harsh Reservoir Conditions

Cheng Huang[1,2*], Hongyi Li[1,2], Xuefeng Gong[1,3]

1.School of Energy, China University of Geosciences (Beijing), Beijing, China, 100000

2.Shengli Oilfield Oil Development Center Co., LTD, Dongying, China, 257100

3. Academy of Science and Technology, China University of Petroleum (East China), Dongying, China, 257061

Corresponding author: Cheng Huang, Email: huangcheng830201@163.com

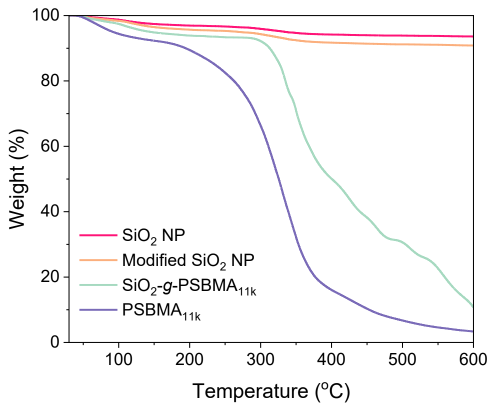

Figure S1. TGA curves of original SiO_2 NP, modified SiO_2 NP, resulting SiO_2-*g*-$PSBMA_{11k}$ and $PSBMA_{11k}$ polymer under N_2 atmosphere.

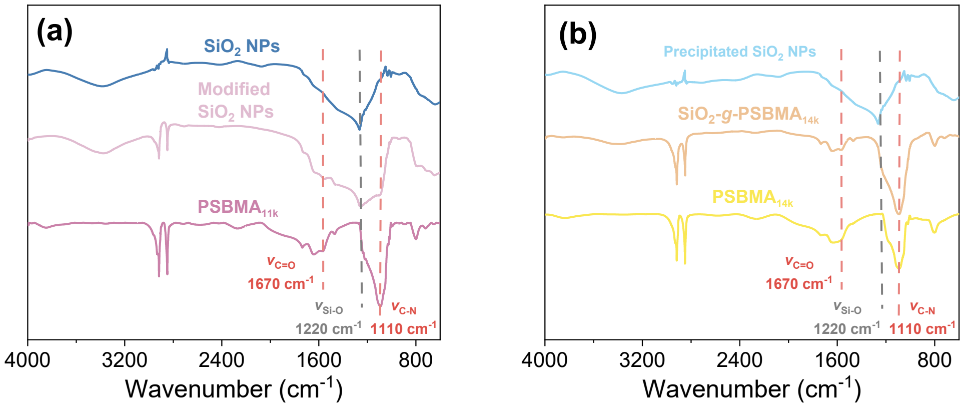

Figure S2. (a) FTIR spectra of original SiO_2 NP, modified SiO_2 NP and $PSBMA_{11k}$ polymer. (b) FTIR spectra of original SiO_2-*g*-$PSBMA_{14k}$ before treatment and precipitated SiO_2 NP and dried $PSBMA_{14k}$ polymer after TBAF treatment.

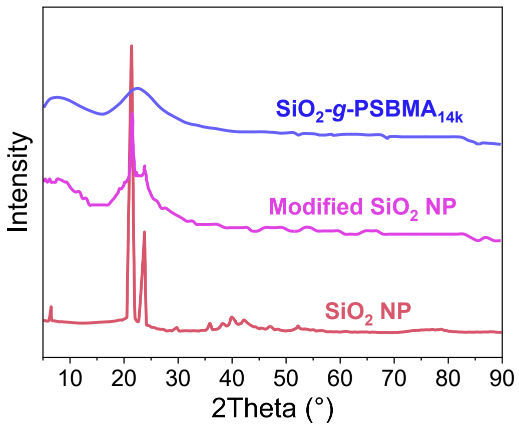

Figure S3. WAXD patterns of original SiO_2 NP, modified SiO_2 NP and resulting SiO_2-*g*-$PSBMA_{14k}$.

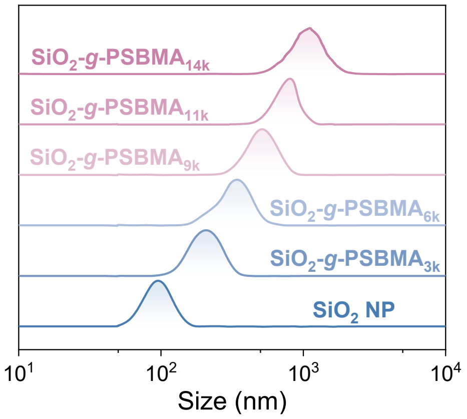

Figure S4. DLS analysis of original SiO_2 NP and SiO_2-*g*-PSBMA hybrids with various $f_{SiO2\ NP}$ at the concentration of 1 mg mL^{-1}.

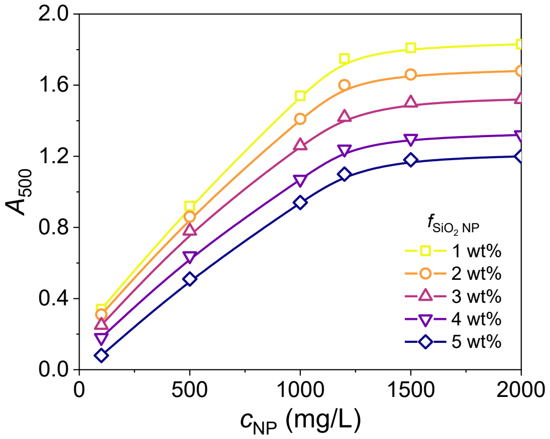

Figure S5. Rises in A_{500} with increasing c_{NP} of SiO_2-*g*-PSBMA hybrids at various $f_{SiO2\ NP}$.

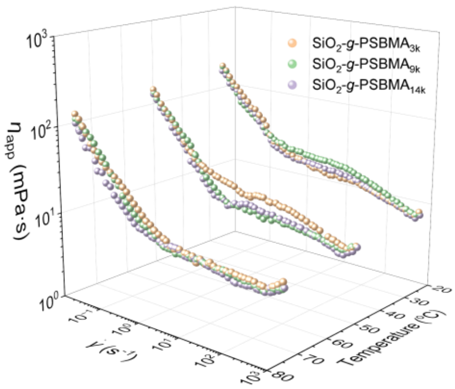

Figure S6. Plots of η_{app} of various SiO_2-*g*-PSBMA aqueous solutions (1 g L^{-1}) and $\dot{\gamma}$ at various temperature.

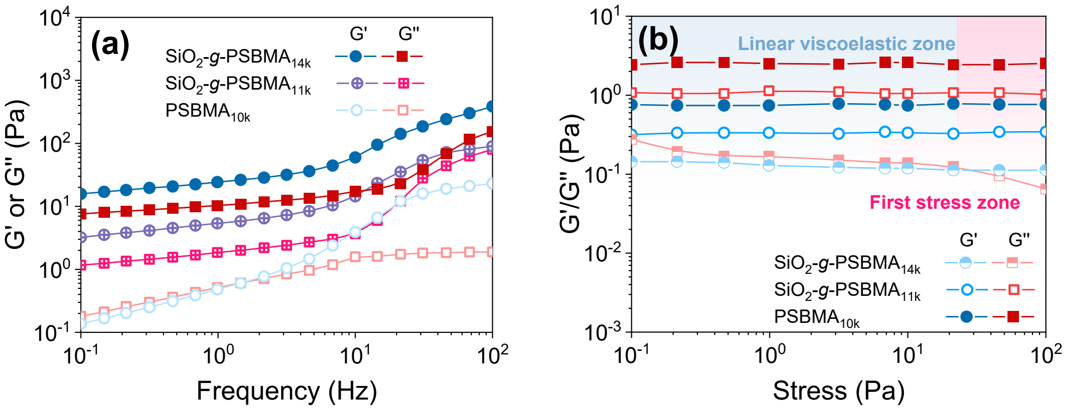

Figure S7. Oscillatory shear rheology of SiO_2-g-PSBMA hybrids and neat PSBMA at 25 °C. (a) Frequency sweep at a fixed strain of 1% showing storage modulus (G′, circles) and loss modulus (G″, squares) as functions of angular frequency. (b) Stress sweep at a fixed frequency of 1 Hz showing G′ and G″ as functions of shear stress; the linear viscoelastic zone and first stress zone are indicated.

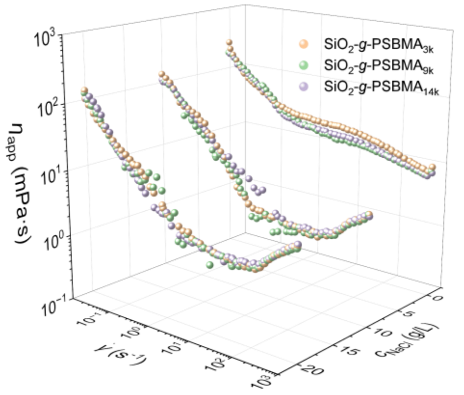

Figure S8. Plots of η_{app} of various SiO_2-*g*-PSBMA (1 g L^{-1}) and $\dot{\gamma}$ with various c_{NaCl} at 30 ºC.

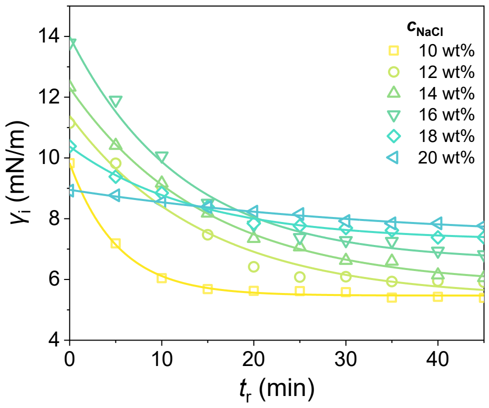

Figure S9. Decreases in interface tension (γ_i) on the interface between SiO_2-*g*-$PSBMA_{14k}$ solutions (1 g

L^{-1}) and core samples with prolonging recovering time (t_r) and increasing c_{NaCl} at 90 °C.

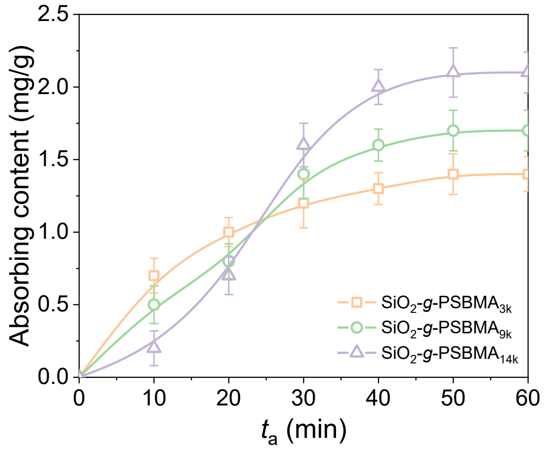

Figure S10. Relationships between absorbing contents of various SiO_2-*g*-PSBMA solutions on dry core samples with various c_{NP} at 90 °C (c_{NP} = 1.0 g L^{-1}, $D = 52 \times 10^3$ μm^2, V_{in} = 0.2 mL min^{-1}).

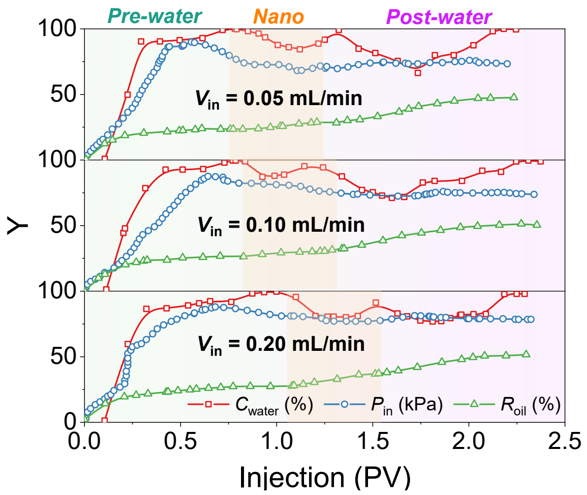

Figure S11. Changes in water content (C_{water}), injection pressure (P_{in}) and recovery rate (R_{oil}) of SiO_2-*g*-$PSBMA_{14k}$ fracturing fluid with various V_{in} at 90 °C (permeability, $D = 52 \times 10^3$ μm^2, flow velocity, c_{NP} = 1 g L^{-1}).
